# Unraveling the complexity of vascular tone regulation: a multiscale computational approach to integrating chemo-mechano-biological pathways with cardiovascular biomechanics

**DOI:** 10.1007/s10237-024-01826-6

**Published:** 2024-03-20

**Authors:** Michele Marino, Bastien Sauty, Giuseppe Vairo

**Affiliations:** 1 Department of Civil Engineering and Computer Science Engineering, University of Rome Tor Vergata, Via del Politecnico 1, 00133 Rome, Italy; 2 Mines Saint-Etienne, Université Jean Monnet, INSERM, U1059 SAINBIOSE, F-42023 Saint-Etienne, France

**Keywords:** Cardiovascular biomechanics, Chemo-mechano-biological modeling, Multiscale computational approach, Vascular tone regulation, Nitric oxide signaling, Adaptive homeostasis

## Abstract

Vascular tone regulation is a crucial aspect of cardiovascular physiology, with significant implications for overall cardiovascular health. However, the precise physiological mechanisms governing smooth muscle cell contraction and relaxation remain uncertain. The complexity of vascular tone regulation stems from its multiscale and multifactorial nature, involving global hemodynamics, local flow conditions, tissue mechanics, and biochemical pathways. Bridging this knowledge gap and translating it into clinical practice presents a challenge. In this paper, a computational model is presented to integrate chemo-mechano-biological pathways with cardiovascular biomechanics, aiming to unravel the intricacies of vascular tone regulation. The computational framework combines an algebraic description of global hemodynamics with detailed finite element analyses at the scale of vascular segments for describing their passive and active mechanical response, as well as the molecular transport problem linked with chemo-biological pathways triggered by wall shear stresses. Their coupling is accounted for by considering a two-way interaction. Specifically, the focus is on the role of nitric oxide-related molecular pathways, which play a critical role in modulating smooth muscle contraction and relaxation to maintain vascular tone. The computational framework is employed to examine the interplay between localized alterations in the biomechanical response of a specific vessel segment—such as those induced by calcifications or endothelial dysfunction–and the broader global hemodynamic conditions—both under basal and altered states. The proposed approach aims to advance our understanding of vascular tone regulation and its impact on cardiovascular health. By incorporating chemo-mechano-biological mechanisms into in silico models, this study allows us to investigate cardiovascular responses to multifactorial stimuli and incorporate the role of adaptive homeostasis in computational biomechanics frameworks.

## Introduction

The physiological behavior of the cardiovascular system is governed by various factors, such as blood flow conditions, vessel mechanical response, and smooth muscle cell tone (Quarteroni et al., 2016; Green et al., 2017; Marino, 2019). Non-physiological events affect mechanobiological stimuli applied to vessel cells. This initiates a series of signaling pathways that can result in alterations of tissue behavior at localized sites within vessels tree, impacting local hemodynamic conditions and possibly the overall response of the cardiovascular system. Changes in vessel response can contribute to the restoration of homeostasis, but they may prove inadequate and contribute to the progression of diseases (Lacolley et al., 2017; Loerakker and Ristori, 2020).

In this context, this work addresses the key role played by vascular tone regulation. Vascular tone refers to the degree of constriction or dilation of blood vessels, a process regulated by the contraction or relaxation of vascular smooth muscle cells (SMCs) within the vessel walls. Changes in vascular tone play a critical role in cardiovascular health and the functioning of the circulatory system (Marti et al., 2012). At a global level, vascular tone governs blood flow and pressure by modulating resistance and compliance across all vessel segments—a topic extensively discussed in the literature (Green et al., 2017; Leloup et al., 2019). Yet, the local effects of vascular tone regulation are often overlooked. Variations in SMC contraction significantly impact the local biomechanical state of vessels. Initially, alterations in the vessel’s internal radius affect wall shear stresses, known to be influential in the development of cardiovascular diseases (Gallo et al., 2016; Mazzi et al., 2022). Additionally, SMC contraction finely tunes hemodynamic loads affecting blood pressure and modifies the tissue’s constitutive response, thereby influencing intramural stresses and strains within the vessel wall. These are known to be instrumental in long-term growth and remodeling mechanisms (Cyron and Humphrey, 2017).

Knowledge of the molecular pathways governing the contraction and relaxation of SMCs in the arteries has been greatly enhanced through extensive research (Lacolley et al., 2017; Russell and Watts, 2000). These studies have shed light on the local, humoral, mechanical, and neurogenic regulatory mechanisms involved. It has been established that dysregulation of these pathways can lead to various pathologies, such as hypertension and arterial stiffening (Marti et al., 2012; Sena et al., 2018; Leloup et al., 2019). Such mechanisms occur all along the arterial tree, involving both elastic and muscular arteries (Green et al., 2017; Leloup et al., 2019). A major actor in the maintenance of a functional vascular tone is played by the endothelium, that is the mono-layer of cells covering the inner surface of blood vessels. The endothelium, in a healthy state, functions as a dynamic organ that maintains vascular tone by carefully balancing the production of vasodilators and vasoconstrictors in response to various stimuli (Sena et al., 2013, 2018). Nitric oxide (NO), which serves as the primary mediator of normal vascular function, is released by the endothelium and diffuses within the vessel wall, resulting in the dilation of smooth muscle (Liu et al., 2008; Hall and Garthwaite, 2009; Zhao et al., 2015). For instance, acute changes in blood flow conditions are detected by endothelial cells. They detect variations in shear stress applied by the blood flow through intra-lumen receptors and promptly release NO in response (Mashour and Boock, 1999; Andrews et al., 2010). The rapid release of NO leads to alterations in vascular tone (Green et al., 2017).

However, the precise physiological mechanisms governing the regulation of SMC contraction and relaxation remain uncertain. The complexity of vascular tone regulation arises from its multiscale and multifactorial nature. Global hemodynamic conditions, such as heart rate and resistance in downstream vasculature, have a significant impact on local flow conditions, local pressure fields, and tissue stresses, that in turn affect the biochemical pathways that influence vascular tone. Conversely, vascular tone influences vessel resistance and compliance, which in turn determine global and local hemodynamic conditions. Although numerous well-established *in vitro* studies have provided evidence on the regulation of SMC contraction and relaxation by shear stress and NO since the early 1990s (Rees et al., 1989; Buga et al., 1991; Kuchan and Frangos, 1994; Corson et al., 1996; Mashour and Boock, 1999; Qiu et al., 2001; Andrews et al., 2010; Wang et al., 2023a), understanding their significance in in vivo cardiovascular conditions and translating this knowledge into clinical practice remains a major unresolved challenge.

Computational models have proven to be highly effective in accurately describing various aspects of cardiovascular biomechanics, providing insights that are difficult to obtain through experimental means. Without the aim of being exhaustive, Table 1 lists several notable examples from the literature, providing context for positioning the present work in the current state of the art.

**Table 1:** Positioning of present work with respect to selected papers on the biomechanics of vessel segments: a focus on multiscale descriptions of the cardiovascular system and on chemo-mechano-biological tissue models

Reference	Vessel geometry	Tissue mechanics	Biochemical signaling	Blood flow (multiscale coupling)	Solution method	Goal of the study
Ismail et al. (2013)	3D (AA)	Hyperelastic	−	FSI (0D-3D)	FEM (PDEs)	Patient-specific circulation model
Polzer and Gasser (2015)	3D (AA)	Hyperelastic (with residual strains)	−	−	FEM (PDEs)	AA Risk Index
Aparício et al. (2016)	Ideal cylinder	G&R	Proteases and growth factors	−	Quasi-analytical (ODEs)	Biochemical model of G &R
Bianchi et al. (2017)	3D (AA)	Hyperelastic	−	FSI (0D-3D)	FEM (PDEs)	AA Risk Index
Marino et al. (2017)	Ideal cylinder	G&R	Proteases and growth factors	−	Quasi-analytical (ODEs)	Biochemical model of G &R
Murtada et al. (2017)	Ideal cylinder	Active	Myosin motor model	−	Quasi-analytical (ODEs)	Arterial contractility
Wilstein et al. (2018)	Ideal cylinder	Active, G&R	NO-ROS pathway, proteases and growth factors	−	Quasi-analytical (ODEs)	Hypertension-induced G &R
Mousavi et al. (2021)	3D (AA)	G&R	−	FSI (0D-3D)	FEM (PDEs)	AA progression
Irons et al. (2022)	Ideal cylinder	Active, G&R	Receptors, proteases and growth factors	−	Quasi-analytical (ODEs)	Biochemical model of homeostasis
Gierig et al. (2023)	Flat tissue	Damage, G&R	Proteases and growth factors	−	FEM (PDEs)	Damage-induced arterial inflammation
Present work	2D (generic)	Active	NO-ROS pathway	ROM (0D-2D)	FEM (PDEs)	Blood flow-related vasodilation

*G&R* Growth and remodeling; *PDEs* Partial differential equations; *ODEs* Ordinary differential equations; *AA* Aortic aneurysm; *FSI* Fluid–structure interaction; *ROM* Reduced-order model

*In silico* models focusing on arterial segments at the scale of millimeters to centimeters have successfully captured the biomechanical state of tissues (Gasser et al., 2010; Polzer and Gasser, 2015; Bianchi et al., 2017; Horvat et al., 2019; Geith et al., 2020; Mousavi et al., 2021) and the local–global hemodynamic conditions (Greve et al., 2006; Ismail et al., 2013; Quarteroni et al., 2016; Romarowski et al., 2018; Kumar et al., 2018; Kim et al., 2021; Mazzi et al., 2022). These models account for detailed characteristics of arterial structures at specific locations within the vessel tree, potentially even incorporating patient-specific information. However, the integration of such biomechanical descriptions with chemo-biological pathways remains limited to a few examples (Aparício et al., 2016; Marino et al., 2017; Irons et al., 2022; Uhlmann and Balzani, 2023; Gierig et al., 2023). Simultaneously, mathematical models focusing on the molecular biology of NO and its regulation by shear stress stimuli have been developed (Buerk et al., 2003; Lamkin-Kennard et al., 2004; Kang et al., 2007; Sriram et al., 2012) that led to a deeper understanding of NO reaction kinetics in vessels. However, these descriptions primarily operate at the molecular length scale and are not effectively integrated with upper-scale models of arterial biomechanics.

This study introduces an *in silico* model of vascular tone regulation through NO-related molecular pathways in response to shear stresses. To the best of the authors’ knowledge and as highlighted in Table 1, state-of-the-art approaches often provide an overly simplified representation of arterial biomechanics (Lanzarone et al., 2009; Wang et al., 2017; Liu et al., 2018; Wilstein et al., 2018; Moshfegh et al., 2021) or do not account for inter-cellular molecular pathways (Schmitz and Böl, 2011; Stålhand et al., 2011; Böl et al., 2012; Murtada et al., 2017). They also generally lack integration with the cardiovascular system, severe limitation considering that the global hemodynamics affects the actual loads applied to the single vascular segments (internal pressure and shear stresses). Hence, their applicability in realistic scenarios is limited since under-representing the complexity of chemo-mechano-biological coupling. In contrast, this paper presents a computational framework that enables a two-way coupling between:

1. global hemodynamic features, modeled using reduced-order approaches;
2. a refined description of passive and active biomechanical behaviors of arterial tissues through detailed finite element analyses;
3. and chemo-biological pathways that account for diffusion–reaction mechanisms regulated by local hemodynamic conditions.

The computational framework is employed to examine the interplay between localized alterations in a specific vessel segment—such as those induced by calcified portions or endothelial dysfunction—and the broader global hemodynamic conditions—both under basal and altered states. The investigations prove the suitability of the developed framework in clarifying the impact of these interactions on the regulation of vascular tone through the NO-ROS pathway. Specifically, it aims to understand how these interactions alter the local biomechanical state of the vessel of interest in terms of wall shear stresses and intramural stresses/strains.

**Fig. 1:**
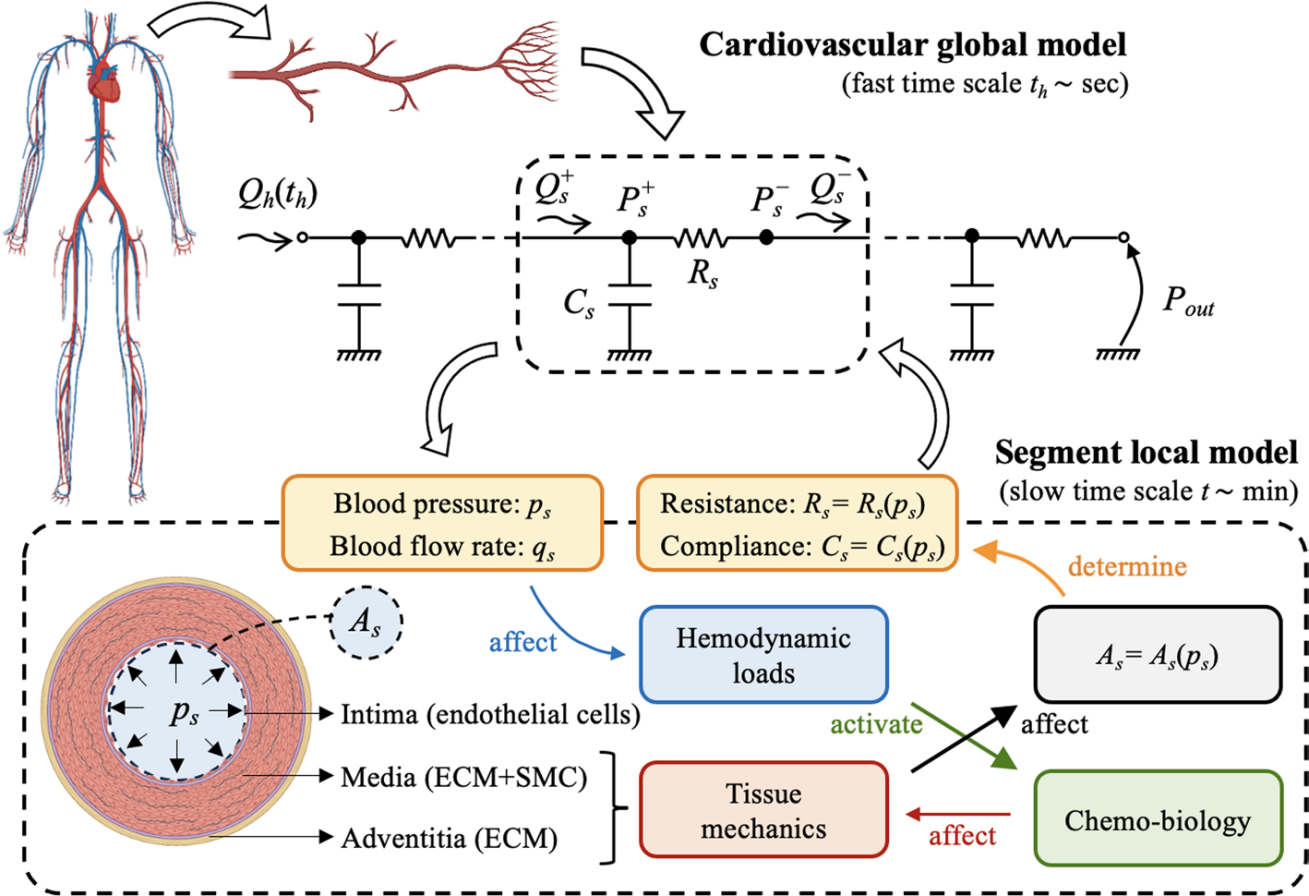
Multi-scale and multi-field approach developed for the computational modeling of cardiovascular biomechanics problems. The global cardiovascular tree is modeled via a 0D description, where each segment is modeled through a resistance $R_s$ and compliance $C_s$. Upstream and downstream pressures $P_s^{+/-}$ and flows $Q_s^{+/-}$ vary in time over a fast time scale $t_h$ that pertains over cardiac cycles. At the local level, a detailed chemo-mechano-biological model is introduced for the segment of interest, using the resulting pressure–radius relationship to determine values of $R_s$ and $C_s$ and how these are influenced by both chemical and mechanical stimuli. These alterations occur gradually over a slower time scale *t*, primarily associated with biochemical changes

## Materials and Methods

A schematic representation of the workflow proposed in this study is depicted in Fig. 1. The framework encompasses both mechanical and chemo-biological equilibrium conditions. The mechanical aspect includes hydrodynamic equilibrium of blood flow and balance of linear momentum within vessel tissues and structures. On the other hand, the chemo-biological aspect involves mass balance equations.

These phenomena operate on distinct time scales. Mechanical equilibrium conditions fluctuate with each heartbeat, typically occurring within seconds, while chemo-biological processes are influenced by molecular production, degradation, and changes in cellular activities, taking place over hours or longer. Therefore, it is advantageous to introduce two time scales (Gharahi et al., 2023):

- a slow time scale that pertains to biochemical alterations and is represented by the time variable *t*. The slow time scale encompasses the duration from 0 to *T*, where *T* represents the total duration of the observed biochemical alteration. The time range $t \in [0, T]$ is discretized into $N-1$ discrete time intervals, resulting in the identification of *N* time values denoted as $t_k$ (where $k = 1, \ldots , N$). The characteristic time *T* of biochemical alterations is typically measured in hours, thereby making the time intervals $\delta t_k = t_k - t_{k-1}$ in the order of minutes.
- a fast time scale that pertains to cardiac cycles and operates over the time variable $t_h$, where the subscript *h* denotes heartbeats. Denoting by $T_h$ the duration of a cardiac cycle (from the beginning of systole to the conclusion of diastole), a sequence of $n_h$ cardiac cycles occurs within the time interval $t_h \in [t, t + n_h T_h]$. Here, *t* represents a given time in the slow time scale, denoting the initiation of the first heartbeat in the series. The overall time interval is discretized into a collection of $N_h$ discrete values, denoted as $t_{h,i}$ (where $i = 1, \ldots , N_h$). The characteristic time $n_h T_h$ for a series of heartbeats is typically measured in seconds, resulting in time intervals $\delta t_{h,i} = t_{h,i} - t_{h,i-1}$ that are fractions of a second.

Following the principles of global–local approaches in continuum mechanics (Gerasimov et al., 2018), the proposed framework is founded upon the definition and coupling of two models designed for significantly different length scales. The global model encompasses (a portion of) the cardiovascular system, and it is introduced in Sect. 2.1. In contrast, the local model focuses on the behavior of an individual vessel segment and includes a detailed description of arterial tissue behavior that establishes the link between its biochemical state and mechanical response. The local model is outlined in Sect. 2.2. The coupling strategy that bridges the global and local models is discussed in Sect. 2.3. Model specialization for a specific case study, along with information on the numerical formulation, is provided in Sect. 2.4.

### Global model: cardiovascular system

The cardiovascular system is macroscopically described as a discrete network of vascular segments (identified by subscript *s*). Each segment is considered a right cylindrical structure with a length $\ell _s$ and a lumen cross-sectional area $A_s$. The quantities of interest are the blood flow and pressure drop across each segment, spanning from the inlet to the outlet. These quantities are evaluated for a given input blood flow law $Q_h(t_h)$ originating from the heart, assumed to be periodic in the fast time scale $t_h$ over an heartbeat duration $T_h$, and a given outflow pressure $P_{out}$ downstream to the network.

Due to the aggregative nature of the quantities of interest, a lumped parameter description is adopted, wherein each segment is represented by a 0D model, as illustrated in Fig. 1 (Korakianitis and Shi, 2006; Quarteroni et al., 2016). Within each segment, the fluid problem takes into account both the frictional loss of blood flow and the elasticity of vascular tissues. The latter factor influences changes in the cross-sectional area $A_s$ of the lumen, which vary with alterations in the internal blood pressure within the *s*-th segment. The mean pressure within the segment, denoted as $p_s$, is chosen as the governing variable, leading to $A_s = A_s(p_s)$.

The frictional and tissue elastic mechanisms are characterized by the introduction of the resistance $R_s$ and the compliance $C_s$ of the *s*-th vascular segment. As described for instance by Quarteroni et al. (2016), these quantities are functions of the cross-sectional area $A_s$, and consequently the pressure $p_s$, via:

1 $$\begin{aligned} R_s(p_s) = \frac{\rho _b K_R \ell _s}{\big (A_s(p_s)\big )^2}\,, \qquad C_s(p_s) = \ell _s \frac{d A_s}{d p_s}\,, \end{aligned}$$

where $\rho _b$ is blood density and $K_R$ is a friction parameter. The latter is related to the kinematic blood viscosity $\mu _b$ and assumes a specific form depending on the blood velocity profile at hand. In what follows, a Newtonian fluid and a parabolic Poiseuille profile are assumed, yielding $K_R=8\pi \mu _b$, (Quarteroni et al., 2016).

For each segment *s*, the pressure and flow rate at the upstream end are denoted by $P_{s}^{+}$ and $Q_{s}^{+}$, respectively, while the downstream quantities as $P_{s}^-$ and $Q_{s}^-$ (see Fig. 1). Since the input blood flow to the system $Q_h$ varies with the fast time scale, the same occurs for upstream/downstream quantities $P_{s}^{{+}/{-}}=P_{s}^{{+}/{-}}(t_h)$ and $Q_{s}^{{+}/{-}}=Q_{s}^{{+}/{-}}(t_h)$. By neglecting convective terms, upstream and downstream pressures and blood flows for each segment are related through the mass conservation law:

2 $$\begin{aligned} C_s \frac{d P_{s}^+}{d t_h} + Q_{s}^- - Q_{s}^+ = 0 \,, \end{aligned}$$

where it results $Q_{s}^- = (P_{s}^{+}-P_{s}^-)/R_s$, as derived from the momentum conservation law by neglecting blood inertia terms, (Quarteroni et al., 2016). Equation (2) leads to a set of ordinary differential equations, which are closed by the blood flow boundary condition (equal to $Q_h(t_h)$) at the entrance of the network and the pressure boundary condition (equal to $P_{out}$) at its exit. Moreover, appropriate algebraic relationships are introduced to describe the network topology, establishing inter-segment equivalences between upstream and downstream flow rates and pressures among different segments (Quarteroni et al., 2016). For instance, if segment $s-1$ is connected without bifurcations to segment *s* (with the stream direction from $s-1$ to *s*), it follows that $Q_{s-1}^-=Q_{s}^+$ and $P_{s-1}^-=P_{s}^+$.

The mean blood pressure $p_s$ and flow rate $q_s$ in each segment are estimated based on the upstream and downstream quantities, respectively, as $p_{s} = (P_{s}^{+} + P_{s}^-)/2$ and $q_{s} = (Q_{s}^+ + Q_{s}^-)/2$. It is important to note that both $p_{s}$ and $q_{s}$ depend on the fast time scale, that is $p_{s} = p_{s}(t_h)$ and $q_{s} = q_{s}(t_h)$. These quantities serve as input for the local model, effectively connecting the two length scales of interest (see Fig. 1).

### Local model: arterial chemo-mechano-biological response

The resistance $R_s$ and compliance $C_s$ of arterial segments in Eq. (1) depend on the functional relationship $A_s(p_s)$. Currently, this relationship is either disregarded or approximated using phenomenological models, (van de Vosse and Stergiopulos, 2011; Alastruey et al., 2012; Epstein et al., 2015), that are difficult to adapt to the chemo-mechano-biological responses of arterial tissues. In this study, this limitation is addressed by obtaining, for the first time, the functional dependency $A_s(p_s)$ through the solution of a local model that incorporates the chemo-mechano-biology of arterial segments.

The following description addresses a reference non-pathological local vessel model, although results will explore also pathological alterations that will be described when specializing the model to specific case studies in Sect. 2.4.1.

#### Geometry and local hemodynamics

In agreement with the compartmental description introduced in Sect. 2.1, the response of the arterial segment is obtained by analyzing the one of a representative cross section when an homogeneous pressure, equal to the mean segment pressure $p_s=p_s(t_h)$, is applied to its internal boundary. This assumption corresponds to neglect the travel time of the blood within the segment.

**Fig. 2:**
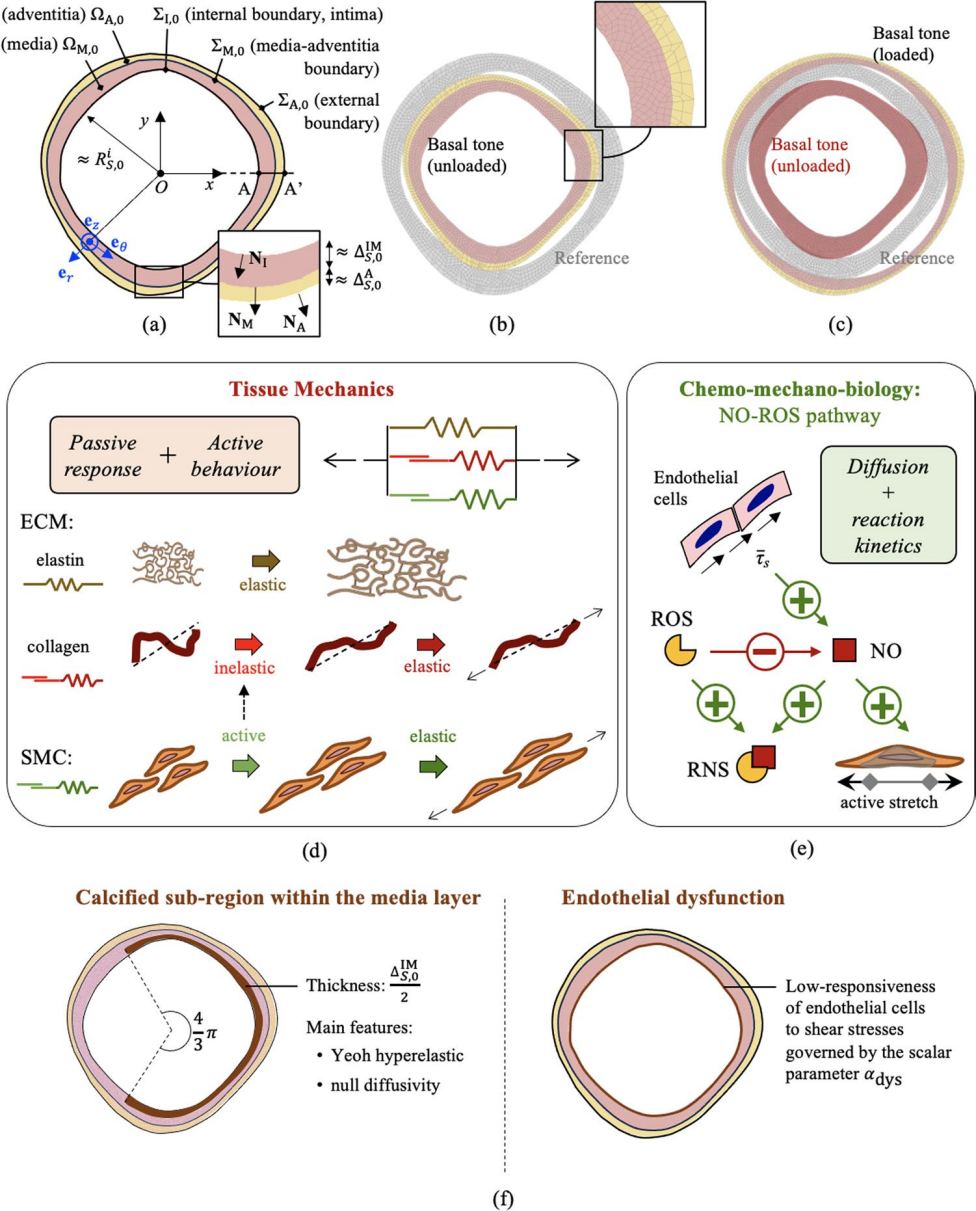
Arterial local model. **a** Reference domain of arterial cross section, with relevant geometrical characteristics (see Sect. 2.2.1). **b**,**c** Reference versus current configurations at basal SMCs active stretch levels $\lambda _{\text {smc}}^{a,b}$, with (b) null pressure and (c) internal pressure of ${100}\,\textrm{mmHg}$ (see Sect. 2.4.1). The finite element mesh adopted in numerical applications is also shown. (d) Tissue constitutive modeling approach (see Sect. 2.2.2). (e) Chemo-mechano-biological modeling approach (see Sects. 2.2.3 and 2.2.4). (f) Alterations considered in the local vessel model as representative of pathological conditions. Acronyms: ECM, extracellular matrix; SMC, smooth muscle cell; NO, Nitric Oxide; ROS, Reactive Oxygen Species; RNS, Reactive Nitrogen Species

As shown in Fig. 2a, arterial cross section is identified by domain $\Omega _0$ in the reference unloaded configuration, in turn split into two non-overlapping sub-domains, that is $\Omega _{\text {M},0}$ associated with the inner media layer, and $\Omega _{\text {A},0}$ with the outer adventitia layer (such that $\Omega _0=\Omega _{\text {M},0}\, \cup \, \Omega _{\text {A},0}$). The internal boundary of $\Omega _{\text {M},0}$ (adjacent to the lumen) identifies the intima layer $\Sigma _{\text {I},0}$, the media-adventitia boundary is identified by $\Sigma _{\text {M},0} = \Omega _{M,0} \, \cap \, \Omega _{\text {A},0}$, while the external vessel boundary of $\Omega _{\text {A},0}$ is denoted by $\Sigma _{\text {A},0}$. All afore-introduced quantities are denoted in the current configuration by omitting the “0” subscript. Moreover, material points within the reference and current configurations are denoted by $\textbf{X} \in \Omega _0$ and $\textbf{x} \in \Omega$, respectively. The normal unit vector to $\Sigma _{\text {I},0}$ (resp., $\Sigma _{\text {I}}$) is denoted by $\textbf{N}_{\text {I}}$ (resp., $\textbf{n}_{\text {I}}$), to $\Sigma _{\text {M},0}$ (resp., $\Sigma _{\text {M}}$) by $\textbf{N}_{\text {M}}$ (resp., $\textbf{n}_{\text {M}}$), and to $\Sigma _{\text {A},0}$ (resp., $\Sigma _{\text {A}}$) by $\textbf{N}_{\text {A}}$ (resp., $\textbf{n}_{\text {A}}$). These unit vectors are all defined outward (i.e., from lumen towards vessel wall).

Arterial cross section is assumed to be quasi-circular, meaning that regions $\Sigma _{\text {I},0}$, $\Sigma _{\text {M},0}$ and $\Sigma _{\text {A},0}$ deviate from perfect circles centered at a common point *O* (representing vessel centerline) by a fraction $\eta < 1$ of the minimum cross-section thickness. To describe the structure of tissue constituents, a cylindrical base system $(\textbf{e}_z, \textbf{e}_r,\textbf{e}_{\theta })$ is introduced in each point within $\Omega _0$, aligned, respectively, with the vessel axial, radial and circumferential directions defined with respect to the position of the vessel centerline *O* (see Fig. 2a). Additionally, since the arterial cross section is quasi-circular, a mean internal radius $r_s^i$ can be conveniently computed from the lumen area $A_s$ as $r_s^i=(A_s/\pi )^{{1}/{2}}$. It is important to note that the arterial cross section and, consequently, the mean radius vary with the fast time scale due to changes in the blood pressure load $p_s(t_h)$, and then $r_s^i=r_s^i(t_h)$.

Following the quasi-circular assumption, the wall shear stresses exerted by blood flow on the arterial wall are estimated by assuming a Poiseuille flow with flow rate $q_s(t_h)$ within an equivalent cylindrical tube having a circular cross section of internal radius $r_s^i(t_h)$. Hence, wall shear stresses $\tau _s$ in segment *s* at time $t_h$ read:

3 $$\begin{aligned} \tau _s(t_h)=\frac{4 \mu _b q_s(t_h)}{\pi \left( r_s^i(t_h)\right) ^3} \,. \end{aligned}$$

Shear stresses trigger the regulation of bio-chemical processes (see Sect. 2.2.3). Such mechanisms have a characteristic time longer than the mechanical process and vary with the slow time scale *t*. Chemical processes at time *t* are assumed to be driven by the time average wall shear stresses (TAWSS) over a series of $n_h$ heartbeats starting at *t*. TAWSS are denoted by $\bar{\tau }_s=\bar{\tau }_s(t)$ and computed as:

4 $$\begin{aligned} \bar{\tau }_s(t)=\frac{1}{n_h T_h}\int _{0}^{n_h T_h} \tau _s(\tau _h + t)\, d\tau _h\,. \end{aligned}$$

#### Arterial tissue mechanics

From the mechanical viewpoint, the local model accounts for the arterial tissue response both in the media and in the adventitia layers. The stiffness of arterial tissues is mainly provided by the passive elastic behavior of the extra cellular matrix (ECM), mainly composed of collagen fibers and elastin (Marino et al., 2021). Moreover, the media layer of arterial tissues is rich of Smooth Muscle Cells (SMCs) that are both passive and active load bearings components. The active response governs the vascular tone, i.e., the state of vasodilation/vasoconstriction. A schematic representation of the main modeling choices is shown in Fig. 2d.

The modeling approach is developed under a large strain framework. For the sake of notation, let $\textbf{F}$ be tissue deformation gradient, $\textbf{C}=\textbf{F}^T\textbf{F}$ the right Cauchy-Green deformation tensor. Arterial tissue mechanics is modeled with an hyperelastic behavior, denoting by $\Psi _{\text {M}}$ and $\Psi _{\text {A}}$ the tissue strain energy densities for the media and adventitia layers, respectively. Two additive (in parallel) contributions to $\Psi _{\text {M}}$ are introduced, representing contributions for the ECM ($\Psi _{\text {ecm}}$) and the SMC ($\Psi _{\text {smc}}$). Moreover, arterial tissues are assumed to be quasi-incompressible, i.e., characterized by a very large value of their bulk modulus $\kappa$. Volumetric locking in numerical applications is prevented through the use of an Augmented Lagrangian formulation, (Marino, 2019).

Tissue strain energy densities read, respectively, for the (healthy) media and adventitia, as:

5a $$\begin{aligned} \Psi _{\text {M}}&= \Psi _{\text {ecm}}+\Psi _{\text {smc}}+p(J-1)-\frac{p^2}{2\kappa } \, , \end{aligned}$$

5b $$\begin{aligned} \Psi _{\text {A}}&= \Psi _{\text {ecm}}+p(J-1)-\frac{p^2}{2\kappa } \, , \end{aligned}$$

where *p* is a pressure-like Lagrange multiplier variable and $J=\sqrt{\textrm{Det}\left( \textbf{C}\right) }$ the volume change associated with $\textbf{C}$. Specific modeling choices for $\Psi _{\text {ecm}}$ and $\Psi _{\text {smc}}$ are described next.

*ECM behavior.* The ECM contribution $\Psi _{\text {ecm}}$ is split into an isotropic response, related to the elastin content, and an anisotropic one, associated with collagen fibers. The isotropic component of ECM response is modeled by means of the isochoric part of the right Cauchy-Green deformation tensor $\bar{\textbf{C}} =J^{-2/3}\textbf{C}$, and its associated first invariant $\bar{I}_1=\textrm{Tr}\left( \bar{\textbf{C}}\right)$, (Marino, 2019).

The anisotropic component of ECM mechanics considers two collagen fiber families, helicoidally arranged around the axial direction of the arterial segment. Denoting by $\phi _{\text {c},j}$ the helix angle of the *j*-th collagen fiber family with respect to the circumferential direction, then

6 $$\begin{aligned} \varvec{a}_{\text {c},j}= \cos (\phi _{\text {c},j})\textbf{e}_{\theta } + \sin (\phi _{\text {c},j})\textbf{e}_z \,, \end{aligned}$$

is the unit vector defining collagen fiber orientation in the circumferential-axial plane, from which the structural tensor $\textbf{M}_{\text {c},j}=\varvec{a}_{\text {c},j} \otimes \varvec{a}_{\text {c},j}$ follows. The kinematics of the *j*-th collagen fiber family is assumed to follow the overall tissue deformation gradient $\textbf{F}$, that is multiplicatively split into an elastic $\textbf{F}_{\text {c},j}^e$ and an inelastic $\textbf{F}_{\text {c},j}^s$ contribution. The latter accounts for mechanisms such as inter-fiber sliding, variations in lamellar undulation, or rearrangement of constituents, associated with the tissue’s active response. Therefore, it results (see Fig. 2d):

7 $$\begin{aligned} \textbf{F}=\textbf{F}_{\text {c},j}^e \textbf{F}_{\text {c},j}^s \,. \end{aligned}$$

The inelastic deformation of collagen fibers $\textbf{F}_{\text {c},j}^s$ is assumed to be incompressible and expressed as:

8 $$\begin{aligned} \textbf{F}_{\text {c},j}^s = \lambda _{\text {c},j}^s\textbf{M}_{\text {c},j}+ \frac{1}{\sqrt{\lambda _{\text {c},j}^s}}\left( \textbf{I}-\textbf{M}_{\text {c},j}\right) \,, \end{aligned}$$

where $\lambda _{\text {c},j}^s$ is the inelastic straightening stretch, discussed in following Sect. 2.2.4 in relation to tissue chemical state.

The elastic component of the total right Cauchy-Green deformation tensor of each collagen family reads:

9 $$\begin{aligned} \textbf{C}_{\text {c},j}^e =( \textbf{F}_{\text {c},j}^e )^T\, \textbf{F}_{\text {c},j}^e = (\textbf{F}_{\text {c},j}^s)^{-T}\, \textbf{C}\, (\textbf{F}_{\text {c},j}^e )^{-1}\,, \end{aligned}$$

and the corresponding squared elastic stretch along $\varvec{a}_{\text {c},j}$ reads (with $j=1,2$):

10 $$\begin{aligned} I_{4,j}^e=\varvec{a}_{\text {c},j}\cdot \textbf{C}_{c,j}^e \varvec{a}_{\text {c},j} = \textrm{Tr}(\textbf{C}_{\text {c},j}^e \textbf{M}_{\text {c},j}) \,. \end{aligned}$$

The strain energy density $\Psi _{\text {ecm}}$ of the extracellular matrix is defined as the sum of a Neo-Hookean incompressible isotropic material for the elastin content ($\Psi _{\text {ecm}}^{\text {el}}$) and an exponential strain energy density for collagen fiber families ($\Psi _{\text {ecm}}^{\text {c},j}$), reading:

11 $$\begin{aligned} \Psi _{\text {ecm}}(\bar{I}_1,I_{4,1}^e,I_{4,2}^e)=\underbrace{\frac{\mu }{2}\left( \bar{I}_1 -3\right) }_{\displaystyle \Psi _{\text {ecm}}^{\text {el}}(\bar{I}_1)} +\sum _{j=1}^2 \underbrace{\frac{k_{1,j}}{2k_{2,j}}\left[ \text {exp}\left( k_{2,j}\langle I_{4,j}^e-1\rangle ^2-1\right) \right] }_{\displaystyle \Psi _{\text {ecm}}^{\text {c},j}(I_{4,j}^e)}\,, \end{aligned}$$

where $\mu$, $k_{1,1}$, $k_{1,2}$ and $k_{2,1}$, $k_{2,2}$ are positive-valued model parameters, and $\langle \cdot \rangle$ denotes Macaulay Brackets (i.e., such that $\langle x \rangle = \text {max}(x,0)$).

*SMCs behavior.* The active contraction of arterial tissues is triggered by an inelastic deformation of SMCs caused by the chemical activation of myosin motors. The resulting force transferred to the tissue following such active contraction depends on the elastic response of all the other components within cells and the stiffness of surrounding constituents. In agreement with the state-of-the-art, (Stålhand et al., 2011; Zulliger et al., 2004; Murtada et al., 2017), an active strain formulation is adopted, focused solely on the tissue scale description of such active mechanism. SMCs are assumed to be aligned with the circumferential direction, the corresponding structural tensor reading $\textbf{M}_{\text {smc}}=\varvec{e}_{\theta } \otimes \varvec{e}_{\theta }$.

SMCs total deformation is assumed to coincide with the one of the tissue that is the same total deformation gradient $\textbf{F}$ applies. This is multiplicatively split into an inelastic active component $\textbf{F}_{\text {smc}}^a$ and an elastic one $\textbf{F}_{\text {smc}}^e$, that is (see Fig. 2d):

12 $$\begin{aligned} \textbf{F}=\textbf{F}_{\text {smc}}^e\textbf{F}_{\text {smc}}^a \,. \end{aligned}$$

The active stretch $\textbf{F}_a$ is assumed incompressible and expressed as:

13 $$\begin{aligned} \textbf{F}_a = \lambda _{\text {smc}}^a\textbf{M}_{\text {smc}}+ \frac{1}{\sqrt{\lambda _{\text {smc}}^a}}\left( \textbf{I}-\textbf{M}_{\text {smc}}\right) \,, \end{aligned}$$

where $\lambda _{\text {smc}}$ is a measure of the myosin-actin filament sliding, (Murtada et al., 2017), controlled by the chemical state of the tissue (see Sect. 2.2.3).

The elastic component of the total right Cauchy-Green deformation then reads:

14 $$\begin{aligned} \textbf{C}_{\text {smc}}^e = (\textbf{F}_{\text {smc}}^e)^T\,\textbf{F}_{\text {smc}}^e=(\textbf{F}_{\text {smc}}^a)^{-T}\, \textbf{C} \,(\textbf{F}_{\text {smc}}^a)^{-1}\,, \end{aligned}$$

and the elastic stretch of SMCs along $\varvec{e}_{\theta }$:

15 $$\begin{aligned} \ \lambda _{\text {smc}}^e = \sqrt{\varvec{e}_{\theta } \cdot \textbf{C}_{\text {smc}}^e \varvec{e}_{\theta }}=\left[ \textrm{Tr}\left( \textbf{C}_{\text {smc}}^e \textbf{M}_{\text {smc}}\right) \right] ^{{1}/{2}}\,. \end{aligned}$$

**Fig. 3:**
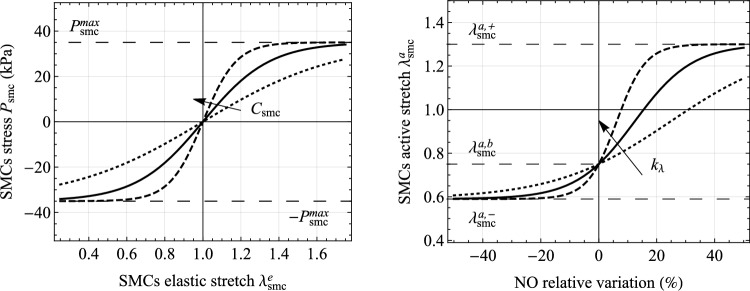
Functions governing the chemo-mechanical response of smooth muscle cells (SMCs). Left: relationship obtained from Eq. (16) between the elastic stretch of SMCs, $\lambda _{\text {smc}}^{e}$, and the scalar measure $P_{\text {smc}}=\partial \Psi _{\text {smc}}/\partial \lambda _{\text {smc}}^e$ of the SMCs’ first Piola-Kirchhoff stress. Right: relationship obtained from Eq. (25) between the relative variation of nitric oxide (NO) concentration, $C_{\text {NO}}$, with respect to the basal concentration, $C_{\text {NO}}^b$, and the active stretch of SMCs, $\lambda _{\text {smc}}^{a}$. The curves are generated using parameter values from Table 2 (continuous lines), with $+100\%$ (dashed line) and $-50\%$ (dotted line) variations of $C_{\text {smc}}$ and $k_{\lambda }$

The strain energy function $\Psi _{\text {smc}}$ is defined as:

16 $$\begin{aligned} \Psi _{\text {smc}}(\lambda _{\text {smc}}^e)= & {} \frac{P_{\text {smc}}^{max}}{C_{\text {smc}}}\biggl\{ P_{\text {smc}}^{max} \log \left[ 1+\text {exp}\left( \frac{2 C_{\text {smc}}}{P_{\text {smc}}^{max}}(\lambda _{\text {smc}}^e-1)\right) \right] \nonumber \\{} & {} -C_{\text {smc}} \lambda _{\text {smc}}^e \biggl\} \,. \end{aligned}$$

where $C_{\text {smc}}$ and $P_{\text {smc}}^{max}$ are two model parameters, representing, respectively, the elastic stiffness of SMCs and the maximum first Piola-Kirchhoff stress during an isometric cell contraction. As shown in Fig. 3, a sigmoid stress-stretch relationship is obtained from Eq. (16).

*Stress–strain relationship and balance laws.* The Cauchy stress tensor $\varvec{\sigma }_{\text {M}}$ of the tissue in the media layer obtained from Eq. (5a) reads:

17a $$\begin{aligned} \varvec{\sigma }_{\text {M}} = J^{-1}{} \textbf{F}\left( 2\frac{\partial \Psi _{\text {M}}}{\partial \textbf{C}} \right) \textbf{F}^T = - p \textbf{I} + {\varvec{\sigma }_{\text {ecm}}} + {\varvec{\sigma }_{\text {smc}}} \end{aligned}$$

resulting in a stress–strain relationship depending on both the ECM tissue response:

17b $$\begin{aligned} {\varvec{\sigma }_{\text {ecm}} = 2J^{-1}{} \textbf{F} \frac{\partial \Psi _{\text {ecm}}^{\text {el}}}{\partial \textbf{C}} \textbf{F}^T + 2J^{-1} \sum _{j=1}^2 \textbf{F}_{\text {c},j}^e \frac{\partial \Psi _{\text {ecm}}^{\text {c},j}}{\partial \textbf{C}_{\text {c},j}^e} (\textbf{F}_{\text {c},j}^e)^T \,,} \end{aligned}$$

and SMCs passive and active behavior:

17c $$\begin{aligned} {\varvec{\sigma }_{\text {smc}} = 2 J^{-1} \textbf{F}_{\text {smc}}^e\frac{\partial \Psi _{\text {smc}}}{\partial \textbf{C}_{\text {smc}}^e} (\textbf{F}_{\text {smc}}^e)^T\,.} \end{aligned}$$

The constitutive relationship for the pressure variable *p* is obtained from the stationary condition of $\Psi _{\text {M}}$ with respect to *p*, that is:

18 $$\begin{aligned} \dfrac{\partial \Psi _{\text {M}}}{\partial p} = 0 \quad \Rightarrow \quad p =\kappa (J-1)\,. \end{aligned}$$

Analogous relationships are obtained for the Cauchy stress tensor $\varvec{\sigma }_{\text {A}}$ from Eq. (5b) for the tissue in the adventitia layer, but here the response is solely passive (i.e., without SMCs contribution and considering $\lambda _{\text {c},j}^s=1$ for all collagen families).

Neglecting body force terms, the balance law of linear momentum gives in the current configuration:

19 $$\begin{aligned}&\text {div}(\varvec{\sigma }_{\text {M}}) = \textbf{0} \quad \text {in }\Omega _{\text {M}} \, ,{} & {} \text {div}(\varvec{\sigma }_{\text {A}}) = \textbf{0} \quad \text {in }\Omega _{\text {A}} \, , \end{aligned}$$

where $\text {div}(\bullet )$ is the divergence operator in the current configuration. The balance of angular momentum gives the symmetry conditions $\varvec{\sigma }_{\text {M}}=\varvec{\sigma }_{\text {M}}^T$ and $\varvec{\sigma }_{\text {A}}=\varvec{\sigma }_{\text {A}}^T$. Equation (19) is completed by the following set of boundary conditions:

20 $$\begin{aligned}&\varvec{\sigma }_{\text {M}}\, \text{n}_{\text {I}} = p_s \text{n}_{\text {I}} \; \text { on }\Sigma_{\text {I}}\, ,{} & {} \varvec{\sigma }_{\text {M}} \, \text{n}_{\text {M}} = - \varvec{\sigma }_{\text {A}} \, \text{n}_{\text {M}} \; \text { on }\Sigma _{\text {M}}\, ,{} & {} \varvec{\sigma }_{\text {A}} \, \text{n}_{\text {A}} = {0} \; \text { on }\Sigma _{\text {A}}\, , \end{aligned}$$

representing the application of the internal lumen pressure $p_s$, the stress continuity at the media-adventitia boundary, and null traction at the outer vessel boundary, respectively. In terms of displacements, minimal boundary conditions are prescribed to remove rigid-body motions, and continuity of displacements is prescribed at the media-adventitia boundary.

It is noteworthy that it might be convenient to pull-back Eqs. (19) in the reference configuration, but special attention should be paid to the application of the internal lumen pressure as a follower load (i.e., in the current configuration), for which the reader is referred to standard textbooks (Wriggers, 2008).

#### Chemo-biological model

Cell–cell signaling pathways involving nitric oxide (NO) and reactive oxygen species (ROS) are addressed, as schematically shown in Fig. 2e. ROS family comprises numerous small reactive ions and molecules that are derived from oxygen metabolism, such as hydrogen peroxide (e.g., unstable free radicals such as superoxide ion $$\hbox {O}_2^{\bullet -}$$ and hydroxyl radical $$\hbox {HO}^{\bullet -}$$, generally transported in stable forms such as hydrogen peroxide $$\hbox {H}_2$$$$O_2$$), (Chen et al., 2018). ROS directly scavenges NO, by forming peroxynitrite $$\hbox {ONOO}^-$$, a reactive nitrogen species (RNS), e.g., through:

21 $$\begin{aligned} \text {NO} + \text {O}_2^{\bullet -} \rightarrow {} \text {ONOO}^-\,. \end{aligned}$$

Thus, ROS indirectly exerts vasoconstrictor effects by depletion of the vasodilatory molecule NO. Direct contraction of SMCs through the activation of specific kinases by means of ROS-mediated pathways has been also reported but are here not considered due to lack of sufficient experimental data, (Chen et al., 2018).

NO and ROS are measured in terms of their molar concentration, resulting functions of space within the arterial tissue and time over the slow time scale *t*. Molecular distributions can be measured either as spatial concentrations (moles per unit current volume), denoted by $C_{NO}$ and $C_{ROS}$ for NO and ROS, respectively, or by $C_{\text {NO}}=J c_{\text {NO}}$ and $C_{\text {ROS}}=J c_{\text {ROS}}$ as material concentrations (moles per unit reference volume in the reference configuration). An isotropic diffusion in the spatial configuration is assumed, introducing the diffusion constants $D_{\text {NO}}$ and $D_{\text {ROS}}$ for the two species.

Within both the media and adventitia layers, function $C_{\text {NO}}=C_{\text {NO}}(\textbf{X},t)$ is obtained from the solution of the mass balance equation of NO reading:

22a $$\begin{aligned} \frac{\partial C_{\text {NO}}}{\partial t} = D_{\text {NO}}\text {Div}\big (J \textbf{C}^{-1} \text {Grad} (C_{\text {NO}} )\big )-\eta _{\text {NO}}C_{\text {NO}}-K_{\text {RNS}}C_{\text {NO}} C_{\text {ROS}}+P_{\text {NO}}^b\,, \qquad \text {in } \Omega _{\text {M},0} \text { and } \Omega _{\text {A},0}\,, \end{aligned}$$

while function $C_{\text {ROS}}=C_{\text {ROS}}(\textbf{X},t)$ from:

22b $$\begin{aligned} \frac{\partial C_{\text {ROS}}}{\partial t} = D_{\text {ROS}}\text {Div}\big (J \textbf{C}^{-1}\text {Grad} (C_{\text {ROS}} ) \big )-K_{\text {RNS}}C_{\text {NO}} C_{\text {ROS}}+P_{\text {ROS}}^b \, \qquad \text {in } \Omega _{\text {M},0} \text { and } \Omega _{\text {A},0}\,. \end{aligned}$$

Here, $\eta _{\text {NO}}$ is the (first-order) reaction rate constant of NO scavenging, $K_{\text {RNS}}$ the (second-order) reaction rate constant of RNS production, $P_{\text {NO}}^b$ and $P_{\text {ROS}}^b$ basal production rates for NO and ROS, respectively.

Equation (22) is completed by the following set of boundary conditions:

23a $$\begin{aligned}&C_{\text {NO}} = C_{\text {NO},e} \, ,{} & {} C_{\text {ROS}} = C_{\text {ROS},e} \, ,{} & {} \text {on } \Sigma _{\text {I},0} \, , \end{aligned}$$

23b $$\begin{aligned}&\text {Grad} (C_{\text {NO}} ) \cdot \textbf{N}_{\text {A}} = 0\, ,{} & {} \text {Grad} (C_{\text {ROS}} ) \cdot \textbf{N}_{\text {A}} = 0\, ,{} & {} \text {on } \Sigma _{\text {A},0} \, , \end{aligned}$$

and by a continuity condition of $C_{\text {NO}}$ and $C_{\text {ROS}}$ fields prescribed at the media-adventitia boundary $\Sigma _\text {M}$. In Eqs. (23a), $C_{\text {NO},e}$ and $C_{\text {ROS},e}$ represent endothelial levels of NO and ROS, respectively. These latter are obtained by solving homogeneous and steady-state forms of mass balance equation (22) in the intima layer, when considering endothelial NO and ROS production rates $P_{\text {NO},e}$ and $P_{\text {ROS},e}$, that is:

24a $$\begin{aligned} \left\{ \begin{array}{l} -\eta _{\text {NO}}C_{\text {NO},e}-K_{\text {RNS}}C_{\text {NO},e} C_{\text {ROS},e}+P_{\text {NO},e} = 0 \\ \\ -K_{\text {RNS}}C_{\text {NO},e} C_{\text {ROS},e}+P_{\text {ROS},e} = 0 \end{array}\right. \quad \Rightarrow \quad \left\{ \begin{array}{l} C_{\text {NO},e} = \dfrac{P_{\text {NO},e} - P_{\text {ROS},e} }{\eta _{\text {NO}}}\\ \\ C_{\text {ROS},e} = \dfrac{P_{\text {ROS},e} \eta _{\text {NO}} }{K_{\text {RNS}}(P_{\text {NO},e} - P_{\text {ROS},e})} \end{array}\right. \,. \end{aligned}$$

On the basis of well-established approaches in the field (Lamkin-Kennard et al., 2004; Sriram et al., 2012), a shear-stress-dependent Michaelis-Menten kinetics is introduced for the NO endothelial production rate $P_{\text {NO},e}$. In detail, TAWSS values $\bar{\tau }_s$ are chosen as governing variables (see Eq. (4)), leading to:

24b $$\begin{aligned} P_{\text {NO},e} = P_{\text {NO}}^b + \frac{R_{\text {NO}}^{max} \text {P}_{O_2}}{K_m + \text {P}_{O_2}} (\bar{\tau }_s - \bar{\tau }_{s,b}) \,, \end{aligned}$$

where $R_{\text {NO}}^{max}$ is the maximum rate of NO production, $\text {P}_{O_2}$ is the partial pressure of oxygen, $K_m$ the Michaelis-Menten constant, and $\bar{\tau }_{s,b}$ a reference value of shear stresses in the basal state.

As regards the endothelial ROS production rate, in the lack of experimental evidence, a linear scaling with $P_{\text {NO},e}$ is assumed, that is $P_{\text {ROS},e} = {P_{\text {ROS}}^b} (P_{\text {NO},e}/P_{\text {NO}}^b)$. In this way, it is straightforward to verify from Eq. (24a) that endothelial ROS concentration is always equal to its basal value, that is $C_{\text {ROS},e}=C_{\text {ROS}}^b$.

#### Vascular tone: chemo-biological regulation of active mechanisms

The concentration field of NO within the media layer governs the vascular tone (see Fig. 2e). To this aim, a direct relationship between $C_{\text {NO}}$ (obtained from Eqs. (22)) and SMCs active stretch $\lambda _{\text {smc}}^a$ is introduced (in Eq. (13)). In particular, it is adopted:

25a $$\begin{aligned} \lambda _{\text {smc}}^a{\mathop {=}\limits ^{\downarrow }} \lambda _{\text {smc}}^a(C_{\text {NO}})=a_{\lambda } + 2 b_{\lambda } \left\{ \frac{1}{c_{\lambda } + \text {exp}[- k_{\lambda } (C_{\text {NO}}/C_{\text {NO}}^b-1)]} - \frac{1}{2}\right\} \,, \end{aligned}$$

where $k_{\lambda } > 0$ is a model parameter governing the sensitivity between NO and active stretches variations (i.e., the slope of function $\lambda _{\text {smc}}^a(C_{\text {NO}})$ at basal value $C{\text {NO}}=C_{\text {NO}}^b$), and quantities $a_{\lambda }$, $b_{\lambda }$ and $c_{\lambda }$ are expressed as:

25b $$\begin{aligned}{} & {} a_{\lambda } = \frac{\lambda _{\text {smc}}^{a,b}(\lambda _{\text {smc}}^{a,+} - 3\lambda _{\text {smc}}^{a,-})+\lambda _{\text {smc}}^{a,-}(\lambda _{\text {smc}}^{a,+} + \lambda _{\text {smc}}^{a,-})}{2(\lambda _{\text {smc}}^{a,+} - \lambda _{\text {smc}}^{a,-})}\,,\nonumber \\{} & {} b_{\lambda } = \frac{(\lambda _{\text {smc}}^{a,+}-\lambda _{\text {smc}}^{a,-})(\lambda _{\text {smc}}^{a,b} -\lambda _{\text {smc}}^{a,-})}{2(\lambda _{\text {smc}}^{a,+}-\lambda _{\text {smc}}^{a,b})}\,,\nonumber \\{} & {} c_{\lambda } = \frac{\lambda _{\text {smc}}^{a,b}-\lambda _{\text {smc}}^{a,-}}{\lambda _{\text {smc}}^{a,+}-\lambda _{\text {smc}}^{a,b}}\,. \end{aligned}$$

Here, $\lambda _{\text {smc}}^{a,+}$ represents the maximum active stretch of dilated SMCs at high NO concentrations $C_{\text {NO}} \gg C_{\text {NO}}^b$, $\lambda _{\text {smc}}^{a,-}$ the minimum active stretch of contracted SMCs at low $C_{\text {NO}} \ll C_{\text {NO}}^b$, and $\lambda _{\text {smc}}^{a,b}$ the active stretch of SMCs at basal NO concentrations $C_{\text {NO}}=C_{\text {NO}}^b$, that is:

25c $$\begin{aligned} &\lim _{k_{\lambda }\rightarrow +\infty } \lim _{C_{\text {NO}}\rightarrow +\infty } \lambda _{\text {smc}}^a(C_{\text {NO}}) = \lambda _{\text {smc}}^{a,+}\,, \\ &\lim _{k_{\lambda }\rightarrow +\infty } \lambda _{\text {smc}}^a(0) = \lambda _{\text {smc}}^{a,-} \,,\\ &\lambda _{\text {smc}}^a(C_{\text {NO}}^b) = \lambda _{\text {smc}}^{a,b} \,. \end{aligned}$$

As shown in Fig. 3, a sigmoid relationship between $C_{\text {NO}}$ and $\lambda _{\text {smc}}^a$ is obtained from Eqs. (25).

Inelastic mechanisms associated with the rearrangement of collagen fibers (described by $\lambda _{\text {c},j}^s$ for the *j*-th collagen fiber family, see Eq. (8)) are in turn governed by active stretches $\lambda _{\text {smc}}^{a}$ of SMCs through:

26 $$\begin{aligned} \lambda _{\text {c},j}^s = 1+ \xi _{\text {c}}(\lambda _{\text {smc}}^{a}-1)\,, \quad \forall \, j\,, \end{aligned}$$

where $\xi _{\text {c}} \in [0,1]$ is the fraction of inelastic stretch transferred from SMCs to collagen structures.

### Multiscale coupling approach

At the scale of the local model, consider the chemo-biological state at a given time *t* along the slow-time scale. This state is determined by solving Eqs. (22) and (23) at time *t*, considering a specific endothelial NO production in Eqs. (24) for a given value $\bar{\tau }_s$ of TAWSS. The solution field $C_{\text {NO}}(\textbf{X},t)$ is employed in Eqs. (25) and (26) to obtain distributions of $\lambda _{\text {smc}}^{a}$ and $\lambda _{\text {c},j}^{s}$ within the tissue, respectively. By considering this resulting inelastic strain field, the balance of linear momentum in Eq. (19) is solved to investigate the mechanical behavior of the arterial cross section. Specifically, the internal pressure $p_s$ in Eq. (20) is varied in the range $[0,p_{max}]$ using $n_p$ incremental load steps. At each pressure step, Eq. (19) is solved via the finite element method, and the internal lumen size $A_s$ computed, thus obtaining pairs of values $(p_s,A_s)$. The relationship between $p_s$ and $A_s$ is approximated within the entire range $p_s \in [0,p_{max}]$ using a surrogate model defined as:

27 $$\begin{aligned} A_s(p_s)=\frac{a}{1 + \text {exp}(-b (p_s/c - 1))} + d\,, \end{aligned}$$

where *a*, *b*, *c*, and *d* are constants to be interpolated from numerical pairs $(p_s,A_s)$. A constrained interpolation procedure, enforcing $c>0$, is performed for minimizing the residual between numerical data and surrogate model predictions. Subsequently, using Eq. (27), arterial resistance and compliance are defined as function of the applied pressure and the specific chemo-biological state at time *t* via Eq. (1).

At the global model level, blood flow and pressure in each segment of the cardiovascular system can be then obtained by solving a nonlinear system of ordinary differential equations derived from Eq. (2). The arterial resistance $R_s(p_s)$ and compliance $C_s(p_s)$, which were previously determined based on the specific chemo-mechano-biological state of arterial cross section at time *t* (along the slow time scale), can be reliably used in this solution, given that the global model operates over the fast time scale variable $t_h$. From the solution of the global model, TAWSS $\bar{\tau }_s$ in Eq. (4) can be computed and used to update the chemo-biological state of the local model.

Therefore, the chemo-mechano-biology of arterial cross section and the hemodynamics in each vessel segment is coupled via a two-way nonlinear coupled system: the local chemo-mechano-biological behavior determines the dependency between $A_s$ and $p_s$, and hence the global hemodynamics; in turn, the latter affects the values of $\bar{\tau }_s$, and hence the boundary conditions of the local chemo-biological problem. In the next Sect. 2.4, a specific solution strategy is discussed to tackle this coupled problem, along with additional information on the numerical strategy for solving the single-scale models.

### Case study and Numerical formulation

This section specializes the previously presented model to the specific case study analyzed in numerical applications, providing also information on numerical implementation procedures. The coupled problem is solved by developing an in-house code implemented in Wolfram Mathematica.

**Table 2:** Values of parameters employed for the local model in numerical applications (if not differently specified), together with relevant literature references and criteria. Acronyms: NO, nitric oxide; ROS, reactive oxygen species; RNS reactive nitrogen species; SMCs, smooth muscle cells; TAWSS, time averaged shear stresses

Description	Parameter	Value	Ref./Criterion
Geometry and pre-stretch			
Unloaded reference radius	$R_{S,0}^i$	5.55 *mm*	Calibrated via Sonesson et al. (1997)
Unloaded intima-media thickness	$\Delta _{S,0}^{\text {IM}}$	0.8 mm	Total thickness 1-1.5mm, (Rosero et al., 2011)
Unloaded adventitia thickness	$\Delta _{S,0}^{\text {A}}$	0.4 mm	
Vessel segment length	$\ell _{S}$	30 cm	Imposed
Axial pre-stretch	$\lambda _z$	1.3	Horný et al. (2017)
Mechanical model (Eqs. (5), (11) and (16))			
Elastin shear modulus	$\mu$	10 kpa	Wang et al. (2023b)
Collagen fibers constants	$k_{1,1}=k_{1,2}$ $k_{2,1}=k_{2,2}$	10 kpa 2.5	Wang et al. (2023b)
Collagen fibers angle	$\theta _{\text {c},1}=-\theta _{\text {c},2}$	45°	Wang et al. (2023b)
SMCs stiffness constants	$C_{\text {smc}}$	100 kpa	Calibrated via Sonesson et al. (1997)
SMCs maximum stress	$P_{\text {smc}}^{max}$	100 kpa	Calibrated via Sonesson et al. (1997)
Tissue bulk modulus	$\kappa$	100 MPa	Calibrated †
Chemo-biological model (Eqs. (22) and (24b))			
NO diffusion constant	$D_{\text {NO}}$	848 ${\mu \textrm{m}^{2}/\textrm{s}}$	Liu et al. (2008)
NO natural decay	$\eta _{\text {NO}}$	0.01 s	Estimated from Condorelli and George (2001); Buerk et al. (2003) ¶
NO basal production rate	$P_{\text {NO}}^b$	80 nM/s	Estimated from Andrews et al. (2010) ¶
NO basal concentration	$C_{\text {NO}}^b$	10 nM	Average value between Andrews et al. (2010) and Hall and Garthwaite (2009)
NO maximum production rate	$R_{\text {NO}}^{max}$	75 nM/s	Estimated from Andrews et al. (2010)§
Oxygen partial pressure	$\text {P}_{O_2}$	90 mmHg	Ortiz-Prado et al. (2019)
Michaelis-Menten constant	$K_m$	5.5 mmHg	Converted from Rengasamy and Johns (1996) at 37°
Basal value of TAWSS	$\bar{\tau }_s^b$	1.74 Pa	Obtained as described in Section 2.4.2
RNS production rate	$K_{\text {RNS}}$	4 nMs	Buerk et al. (2003)
ROS diffusion constant	$D_{\text {ROS}}$	848 $\mu {\text{m}}^{2}/{\text{s}}$	$=D_{\rm{NO}}$
ROS basal production rate	$P_{\text {ROS}}^b$	69.9 nM/s	Eq. (28)
ROS basal concentration	$C_{\text {ROS}}^b$	1.74 nM	Eq. (28)
Chemo-mechano-biological coupling (Eqs. (25) and (26))			
Minimum SMCs active stretch	$\lambda _{\text {smc}}^{a,+}$	0.59	Calibrated via Sonesson et al. (1997)
Basal SMCs active stretch	$\lambda _{\text {smc}}^{a,b}$	0.75	Calibrated via Sonesson et al. (1997)
Maximum SMCs active stretch	$\lambda _{\text {smc}}^{a,+}$	1.3	Imposed
NO-stretch sensitivity	$k_{\lambda }$	100	Imposed
Collagen inelastic stretch fraction	$\xi _{\text {c}}$	0.15	Calibrated ‡

† to enforce quasi-incompressibility

¶ reaction rate between NO and guanylate cyclase in the vascular wall.

§ since experimental data in Andrews et al. (2010) indicate a production rate of circa $50-{100\,\mathrm{\mathrm nM/s}}$ for WSS around 1 Pa and

a maximum production rate of around $150-{160}\,\mathrm{nM/s}$.

‡ to have $-30\%$ decrease of the internal radius from the relaxed to the basal state at systolic pressure

#### Local model specialization

The local model represents one segment of interest in the global model, generically denoted by subscript *S*. As previously introduced, vessel response is investigated by analyzing the one of a single cross section. Its geometry is generated following the procedure described in Appendix A, characterized by a mean reference internal radius $R_{S,0}^i$, the mean intima-media thickness $\Delta _{S,0}^{\text {IM}}$, and the mean adventitia thickness $\Delta _{S,0}^{\text {A}}$ (see Fig. 2a). From the kinematic viewpoint, vessel deformation is assumed to occur in the circumferential-radial plane with a constant axial pre-stretch $\lambda _z$ and null axial-circumferential and axial-radial shear strains. The chemo-mechano-biology problem described in Sect. 2.2 is solved via Finite Element Analyses (FEA).

*Finite element formulation.* The finite element formulation employs five-noded quadrilateral elements. The four (topological) corner nodes host 2 degrees of freedom (*dof*) for displacements and 2 *dof* for $C_{\text {NO}}$ and $C_{\text {ROS}}$, while the fifth node is a local auxiliary one and hosts 1 *dof* for the pressure-like Lagrange multiplier *p* (see Eq. (5)). Concentration’s *dof* represents the remainder between actual concentrations $C_{\text {NO}}$ (resp., $C_{\text {ROS}}$) and basal one $C_{\text {NO}}^b$ (resp., $C_{\text {ROS}}^b$), normalized with respect to basal concentrations. Bi-linear Lagrange polynomials are introduced for displacements and concentrations, while a constant interpolation is employed for the pressure-like Lagrange multiplier. To ensure numerical stability, an average concentration $C_{\text {NO}}^{e}$ is computed in each finite element *e*, which is then used to define element-wise constant $\lambda _{\text {smc}}^a$ and $\lambda _{\text {c},j}^s$ according to Eqs. (25) and (26). The element residual and tangent stiffness are derived taking advantage of the symbolic-numeric libraries of the AceGen plug-in, while assembly and nonlinear solution of the resulting finite element algebraic system (implemented via the Newton–Raphson scheme) are performed using the AceFem plug-in. The computational mesh, consisting of approximately 1500 elements, was determined through a sensitivity study (not reported) and is shown in Fig. 2b. Mesh convergence was confirmed by conducting simulations with finer meshes (up to 2$\times$ elements), which resulted in negligible differences (less than 5%) in the model’s outcomes of interest, such as intramural stresses and strains, species concentrations, and predicted wall shear stresses.

**Fig. 4:**
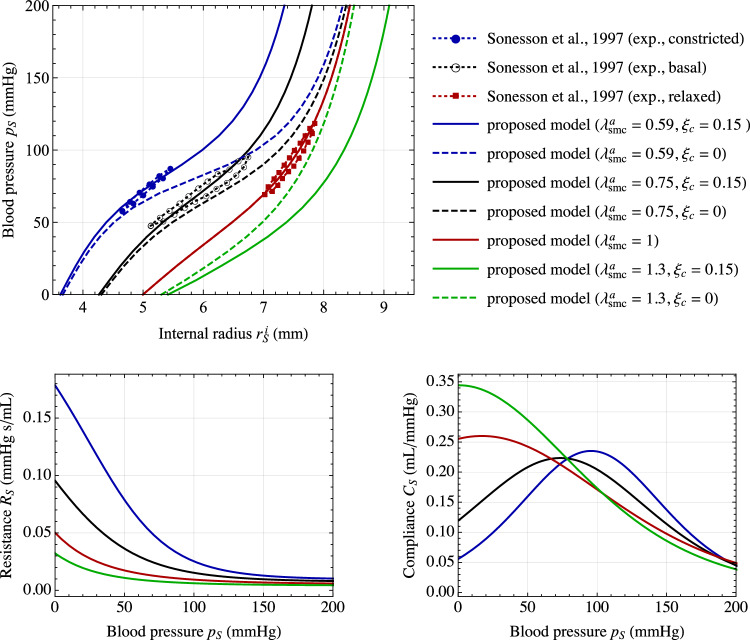
Mechanical response of the arterial local model. Top: pressure–radius curves for different values of active stretches $\lambda _{\text {smc}}^a$, imposed uniformly within the media layer. Results are obtained with ($\xi _{\text {c}}=0.15$) and without ($\xi _{\text {c}}=0$) considering the mechanism of inelastic collagen straightening. Comparison with experimental data by Sonesson et al. (1997) on pressure–radius loops for a human abdominal aorta at three different conditions, i.e., constricted, basal and relaxed. Bottom: corresponding functions $R_S=R_S(p_S)$ and $C_S=C_S(p_S)$, obtained through Eqs. (1) and (27)

*Mechanics.* The vessel cross section introduced in the local model is chosen to be representative of a human abdominal aorta. In fact, geometry and material mechanical parameters are calibrated from experimental data by Sonesson et al. (1997) on pressure–radius loops of a human abdominal aorta at three different conditions: constricted, basal, and relaxed. The values of geometrical and material parameters adopted in all numerical simulations (unless otherwise specified) are listed in Table 2. The pressure–radius relationships obtained for different values of active stretches $\lambda _{\text {smc}}^a$ are reported in Fig. 4, showing an excellent agreement with data by Sonesson et al. (1997) for the three states of vascular tone. In this case, the active stretch is not determined through the chemo-biological coupling (i.e., from Eq. (25)), but is imposed *a priori* as constant values within the media layer. Notably, the basal contraction has a remarkable effect on arterial mechanics, although being a feature often overlooked in current cardiovascular biomechanical models (see Fig. 2b and 2c).

The effects of inelastic collagen straightening are reported in Fig. 4. This is analyzed since, to the best of authors’ knowledge, this effect is introduced in this paper for the first time in the literature. A change in vascular tone leads to a variation of the internal arterial radius at high pressure loads only when considering inelastic collagen straightening mechanisms. This occurs since an inelastic stretch $\lambda _{\text {c},j}^s$ of collagen fibers induces a change in their reference configuration that reduces the total distensibility of collagen fibers for $\lambda _{\text {c},j}^s<1$ (and, vice versa, increases it for $\lambda _{\text {c},j}^s>1$).

The corresponding functions $R_S=R_S(p_S)$ and $C_S=C_S(p_S)$, obtained through Eqs. (1) and (27), are reported in Fig. 4 for different vascular tone states. Remarkably, the obtained values fall within the typical ranges reported in the literature for the resistance and compliance of human vessels (Korakianitis and Shi, 2006; Tossas-Betancourt et al., 2020), and the shape of the function $C_S=C_S(p_S)$ agrees qualitatively with in vivo measurements of arterial compliance versus blood pressure (Kornet et al., 1998), also considering different contraction and relaxation states of SMCs (Bank et al., 1995; Li, 2018).

Additional results on the tissue constitutive relationship derived from Eq. (17) and the performance of the surrogate modeling approach based on Eq. (27) are provided in Appendix B. High-fidelity results for the calibration of the surrogate modeling approach are obtained throughout this study from FEA with $p_{max}={250}\,\textrm{mmHg}$ and $n_p=10$.

*Chemo-biology.* Model parameters governing arterial chemo-biological response (i.e., molecular diffusion and reaction kinetics, as well as how molecular pathways affect vascular tone) are listed in Table 2, chosen from well-established literature references. In particular, since values of molecular basal concentrations and productions rates are closely related, authors decided to set values for NO (i.e., $C_{\text {NO}}^b$ and $P_{\text {NO}}^b$) on the basis of literature evidence, while the corresponding ROS values are obtained in order to satisfy the steady-state and homogeneous version of mass balance equations (22), leading to:

28 $$\begin{aligned} C_{\text {ROS}}^b = \dfrac{1}{K_{\text {RNS}}}\left( \frac{P_{\text {NO}}^b}{C_{\text {NO}}^b}-\eta _{\text {NO}} \right) \,,\qquad P_{\text {ROS}}^b = K_{\text {RNS}} C_{\text {ROS}}^bC_{\text {NO}}^b\,. \end{aligned}$$

The chemo-biological response of the local model alone is analyzed in following Results’ Sect. 3.1.

*Pathological alterations.* Two pathological alterations of the local vessel model will be considered, also highlighted in Fig. 2f:

1. a calcification in the media layer that covers approximately one-third of its total extension This is modeled by considering a strain-energy density function within the calcified sub-region defined via a 3-term quasi-incompressible Yeoh model. Material constants are taken from literature ($c_1=302$ kPa, $c_2=-228$ kPa, and $c_3=261$ kPa, see Buckler et al. (2022)). Moreover, diffusivity of molecular species within the calcified sub-region is considered negligible, (Tzafriri et al., 2017).
2. endothelial dysfunction. This is simulated through a reduced sensibility of endothelial cells to shear stresses. To this aim, the endothelial production rate of NO in Eq. (24b) is re-defined as:
  29 $$\begin{aligned} {P_{\text {NO},e} \rightarrow P_{\text {NO},e}^{\text {dys}} = P_{\text {NO}}^b + (1-\alpha _{\text {dys}})\frac{R_{\text {NO}}^{max} \text {P}_{O_2}}{K_m + \text {P}_{O_2}} (\bar{\tau }_s - \bar{\tau }_{s,b}) \,,} \end{aligned}$$
  where $\alpha _{\text {dys}}$ represents a damage-like variable associated with endothelial dysfunction, taking values $\alpha _{\text {dys}} \in [0,1]$ with $\alpha _{\text {dys}}=0$ representing a sound endothelium (i.e., responsive to shear stresses variations) and $\alpha _{\text {dys}}=1$ a fully damaged (unresponsive) endothelium. In particular, two values of dysfunction will be considered, that is $\alpha _{\text {dys}}=0.5$ for the mild endothelial dysfunction, and $\alpha _{\text {dys}}=0.9$ for the severe one.

**Table 3:** Values of parameters employed for the cardiovascular global model in numerical applications, together with relevant literature references and criteria

Description	Parameter	Value	Ref./Criterion
Resistance of upstream segment	$R_1$	${0.1}\, \mathrm{mmHg\,s/mL}$	Korakianitis and Shi (2006)
Compliance of upstream segment	$C_1$	${0.5}\,{mL/\textrm{mmHg}}$	Korakianitis and Shi (2006)
Resistance of downstream segment	$R_3$	${1}\, \mathrm{mmHg\,s/mL}$	Korakianitis and Shi (2006)
Compliance of downstream segment	$C_3$	${0.001}\,{mL/\textrm{mmHg}}$	Korakianitis and Shi (2006)
Basal heart frequency	$f_1^b$	${1}\,{\text {beats} /\textrm{s}}$	60 Beats per minute (bpm)
Cardiac output	$V_h$	${5.6}\,{\textrm{min}}$	Rusinaru et al. (2021)

**Fig. 5:**
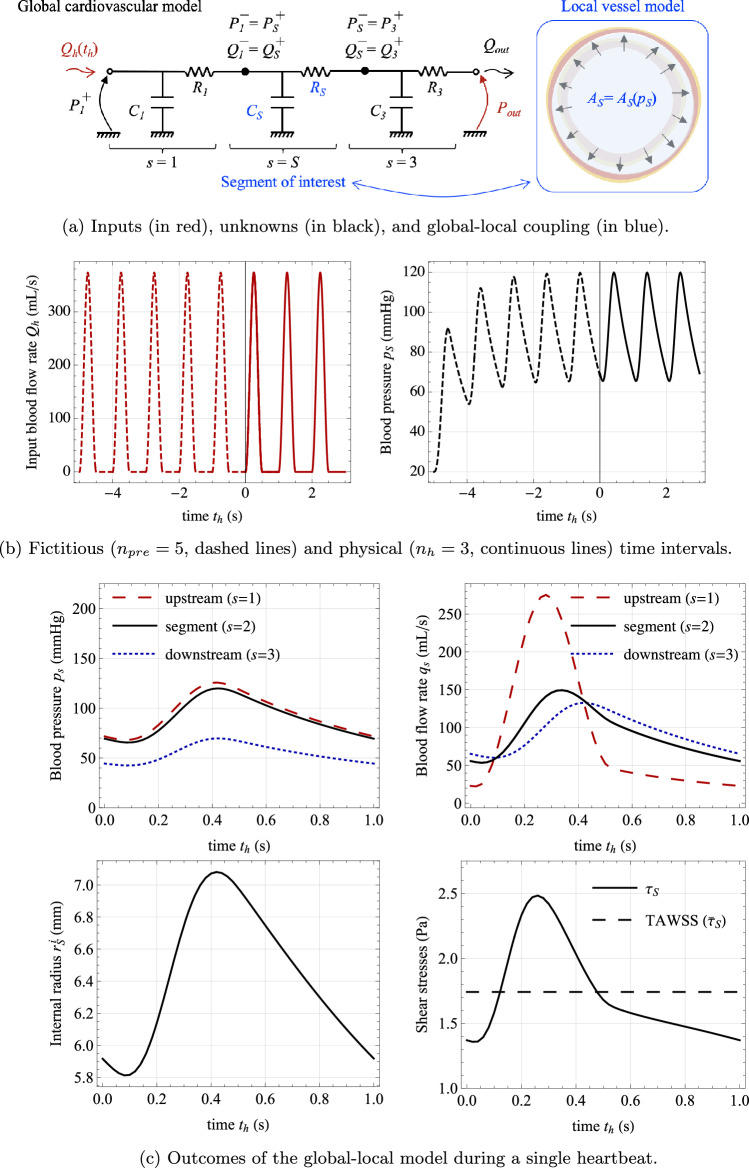
Response of the cardiovascular model. **a** Global-local structure considered in numerical applications. **b** Solution strategy for treating the unknown initial conditions of the system at $t_h=t$. **c** Top: Mean blood pressure $p_s$ and flow rate $q_s$ of the upstream segment ($s=1$), the segment of interest ($s=S=2$) and the downstream vasculature ($s=3$). Bottom: lumen internal radius and shear stresses for the segment of interest. A uniform and basal value of the active stretch $\lambda _{\text {smc}}^a=\lambda _{\text {smc}}^{a,b}$ (see Fig. 4) is considered for computing segment resistance $R_S$ and compliance $C_S$. The values of parameters are given in Tables 2 and 3 and refer to a value for the slow time variable equal to $t=0$

#### Global model specialization

The case study addresses an idealized portion of the cardiovascular system with three non-branching segments, see Fig. 5. The cardiovascular global model consists of an upstream artery ($s=1$), the central arterial segment ($s=2$), and downstream branching capillaries ($s=3$). The chemo-mechano-biological coupling is applied exclusively to the central vessel segment, which is focused in this work. Hence, resistance and compliance of the upstream and downstream segments (i.e., $R_1$, $R_3$, $C_1$, $C_3$) are assumed to be constant, while the ones of the segment of interest (i.e., $R_2=R_S$ and $C_2=C_S$) are obtained from the arterial cross-section local model.

Once that functions $R_S(p_S)$ and $C_S(p_S)$ are available from the local model analyses, the cardiovascular global model can be solved. From Eq. (2) and considering boundary conditions $Q_{1}^+=Q_h(t_h)$ and $P_3^-=P_{out}=\text {const }$, a set of three ordinary differential equations in the fast time scale $t_h$ is obtained:

30a $$\begin{aligned} \textbf{B}_1 \frac{d \textbf{y}}{d t_h} + \textbf{B}_2 \textbf{y} = \textbf{b}\,, \end{aligned}$$

where $\textbf{y}=\textbf{y}(t_h)$ collects the unknown pressures across the different segments and $\textbf{b}=\textbf{b}(t_h)$ flux forcing terms, resulting:

30b $$\begin{aligned} \textbf{y}(t_h) = \begin{pmatrix} P_1^{+} \\ P_S^{+} \\ P_3^{+} \end{pmatrix}\,, \qquad \textbf{b}(t_h) = \begin{pmatrix} Q_h(t_h) \\ 0 \\ {P_{out}}/{R_3} \end{pmatrix}\,. \end{aligned}$$

Moreover, $\textbf{B}_1=\textbf{B}_1(\textbf{y})$ and $\textbf{B}_2=\textbf{B}_2(\textbf{y})$ describe the connectivity of the system and read:

30c $$\begin{aligned} \textbf{B}_1(\textbf{y}) = \begin{bmatrix} C_1 &{} 0 &{} 0 \\ 0 &{} C_S(p_S) &{} 0 \\ 0 &{} 0 &{} C_3 \end{bmatrix}\,, \qquad \textbf{B}_2(\textbf{y}) = \begin{bmatrix} R_1^{-1} &{} - R_1^{-1} &{} 0 \\ - R_1^{-1} &{} R_1^{-1}+ \big (R_S(p_S)\big )^{-1} &{} - \big (R_S(p_S)\big )^{-1} \\ 0 &{} - \big (R_S(p_S)\big )^{-1} &{} \big (R_S(p_S)\big )^{-1}+ R_3^{-1} \end{bmatrix}\,, \end{aligned}$$

with $p_S=(P_S^+ + P_S^-)/2$. Due to network topology, it results $P_1^{-}=P_S^{+}$ and $P_S^-=P_3^{+}$.

The global model solution is sought within the physical time interval $[t, t+n_hT_h]$. However, the initial conditions at $t_h = t$ are unknown. To address this issue, the solving time interval is extended with $n_{pre}$ fictitious preliminary heartbeats (prior to *t*) to ensure a steady-state periodic response within the physical time interval. Thus, Eq. (30) is solved over the time interval $t_h \in [t-n_{pre}T_h, t+n_hT_h]$ with initial conditions at $t_{pre} = t-n_{pre}T_h$ set as $\textbf{y}|_{t_h=t_{pre}}=P_{out}{} \textbf{1}$. A backward Euler discretization is adopted in time, leading to an implicit system of governing equations to be solved. Preliminary numerical tests have demonstrated that a steady-state periodic response is achieved within the physical time interval when $n_{pre}T_h\approx {5\,\mathrm{\text {s}}}$, corresponding to $n_{pre} \in [5,10]$ depending on the heart frequency.

The chosen solution strategy eliminates the need to impose a minimum duration for the physical time interval, as a steady-state periodic response is ensured by the fictitious time extension. However, the duration is still limited from above to preserve the separation of time scales between the slow and fast components. For the subsequent analysis, a value of $n_h=3$ is used.

In numerical applications, the input blood flow $Q_h$ is introduced to mimic the inflow conditions from the heart into the human cardiovascular system. The blood is ejected from the heart with a pulsatile flow of frequency $f_h=1/T_h$. The cardiac pulse consists of two phases: an injection phase lasting $T_h/2$, approximated by a cosine law, and a ventricular filling stage, also lasting $T_h/2$, modeled using a null blood flow condition. It should be noted that the cardiac frequency is assumed to be constant in the fast time scale $t_h$, but may vary with the slow time scale, i.e., $f_h=f_h(t)$. In the solution time interval of the cardiovascular global model, i.e., for $t_h \in [t,t+n_hT_h]$, the input blood flow $Q_h=Q_h(t_h)$ reads:

31a $$\begin{aligned} Q_h(t_h) = \left\{ \begin{aligned}&\dfrac{Q_{a}(t)}{2}\left[ 1-\cos \left( 4\pi f_h(t) (t_h-t)\right) \right]&\quad \text {if} \; t_h \in t+ \left[ n T_h, n T_h + \frac{T_h}{2}\right] \text { for } n\in \mathbb {N}, \, n<n_h\\&0&\textrm{else} \end{aligned} \right. \,, \end{aligned}$$

where $Q_{a}(t)$ represents the flow rate amplitude, constant in the fast time scale $t_h$ but possibly varying with the slow time scale *t*. The flow rate amplitude is here linked to the cardiac frequency $f_h$ such to have a constant cardiac output $V_h$ during a single heartbeat. Therefore, it results:

31b $$\begin{aligned} \int _{0}^{T_h} Q_h(t+ \tau _h)d\tau _h=\dfrac{Q_{a}(t)}{4f_h(t)} {\mathop {=}\limits ^{\downarrow }} V_h\quad \Rightarrow \quad Q_{a} (t) = 4 f_{h}(t) V_h \,. \end{aligned}$$

The parameter values used in the cardiovascular global model are listed in Table 3. Specifically, the values for $R_1$, $C_1$, $R_3$, and $C_3$ are chosen to simulate a distensible and large-caliber upstream vessel for $s=1$ and a non-distensible and small-caliber downstream segment for $s=3$, (Korakianitis and Shi, 2006).

Figure 5 presents results from an exemplary case study, illustrating the response of the introduced cardiovascular global model. The solution strategy, which includes a fictitious and a physical time interval, is also highlighted. This case study focuses on the basal chemo-mechano-biological state of the arterial local model, with boundary conditions $C_{\text {NO},e} = C_{\text {NO}}^b$ and $C_{\text {ROS},e} = C_{\text {ROS}}^b$ in Eqs. (23). Based on the model formulation and parameter settings (see Sect. 2.4.1 and Table 2), a uniform and basal value of SMCs active stretch $\lambda _{\text {smc}}^a=\lambda _{\text {smc}}^{a,b}$ is obtained throughout the media layer. Therefore, from the solution obtained for the cardiovascular global model, the basal TAWSS value $\bar{\tau }_s^b$ in Eq. (24b) is computed using Eq. (4) and employed for the following numerical studies in Sect. 3.

#### Coupling algorithm

In Sect. 2.3, it was emphasized that the coupling between the global and local models results in a nonlinear system of equations. To solve this system, the Aitken-Steffensen’s method is employed, which is an iterative technique for finding the roots of nonlinear functions without requiring the use of the function’s derivative (Johnson and Scholz, 1968; Păvăloiu, 1995).

Let us consider a series of time steps t_1_,...,t_2_ in the slow time scale, and assume that the solution of the coupled system is known up to step (k-1) and the solution at step *k* is sought for. Hence, the actual value of TAWSS, denoted as $\bar{\tau }_s^{(k)}$, is unknown, and $\tau_j$ represents the *j*-th guess within the iterative procedure. By substituting $\bar{\tau }_s = \tau_j$ into Eq. (24b), the corresponding guess value C_j_ of endothelial NO concentration can be determined using Eq. (24a). The chemo-mechano-biological local model is then solved using these values.

The computed segment resistance and compliance values are passed to the cardiovascular global model, resulting in updated TAWSS values denoted as $g(\tau_j)$, where $g(\bullet)$ represents the transfer function of the coupled system. According to the Aitken-Steffensen’s method, the TAWSS guess values are updated at each iterative step using the following scheme:

32 $$\begin{aligned} \tau _{j+1} = \tau _j + \frac{\left( g(\tau _j)- \tau _j\right) ^2}{g(g(\tau _j)) -2g(\tau _j) + \tau _j}\,, \end{aligned}$$

until

33 $$\begin{aligned} {{err}}_j = \frac{ C_{j+1} - C_j}{C_{\text {NO}}^b} < {{tol}} \qquad \Rightarrow \qquad \bar{\tau }_s^{(k)}=\tau _{j+1} \,, \end{aligned}$$

where *tol* is the prescribed tolerance on the error measure *err*. For the subsequent numerical results, a value of ${tol }=10^{-3}$ is chosen. If the error does not decrease over two consecutive iteration steps, i.e., if ${err}_{j+1} > \text {err}_j$, the algorithm is re-initialized by updating the next attempt value as $\tau _{j+1} \rightarrow (\tau _{j+1} + \tau _j)/2$.

**Fig. 6:**
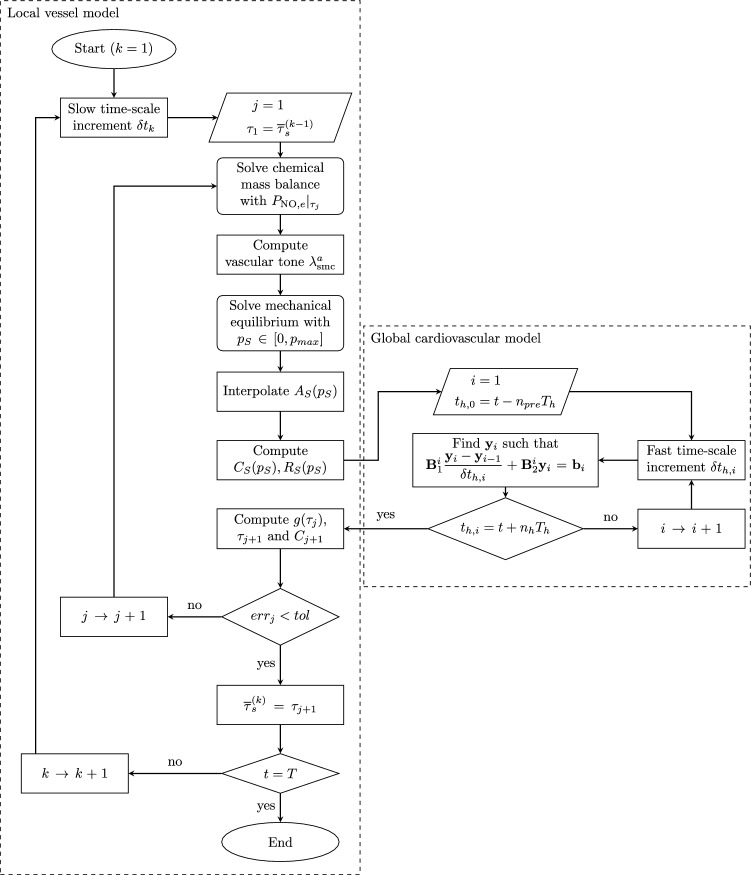
Flowchart of the global–local algorithmic procedure. Initialization values: $\bar{\tau }_s^{(0)}=\bar{\tau }_s^b$ and $\textbf{y}_0=P_{out} \textbf{1}$

It is noteworthy that the iterative scheme in Eq. (32) is initialized at each load step *k* as $\tau _1 = \bar{\tau }_s^{(k-1)}$, that is through the TAWSS value obtained at the previous converged step $(k-1)$. The flowchart of the developed global–local algorithmic procedure is shown in Fig. 6.

## Results

Three numerical applications are considered in the following.

**Fig. 7:**
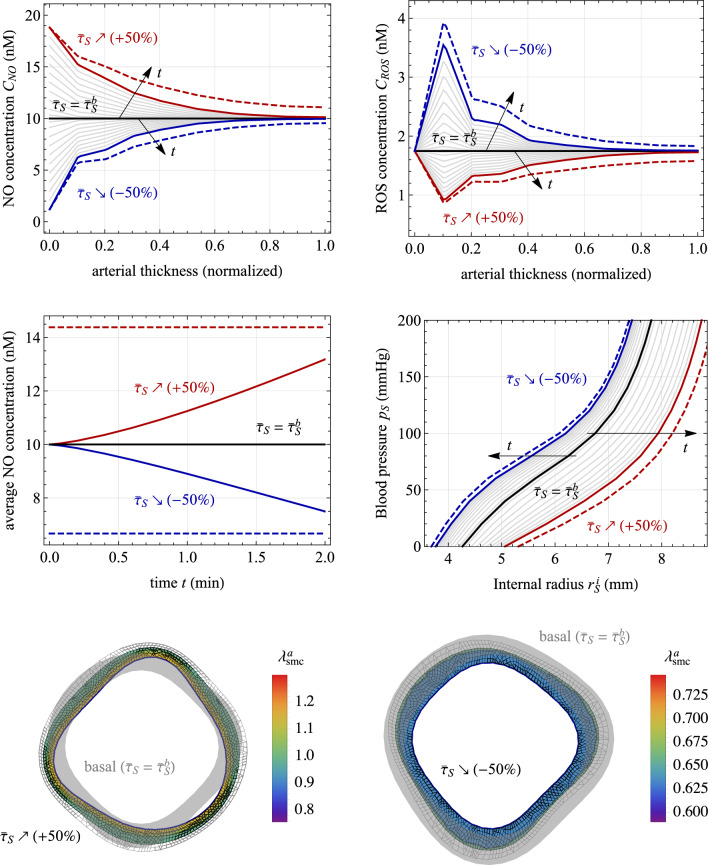
Chemo-mechano-biological response of the arterial local model (Sect. 3.1). Results are obtained for a 2-minute linear-wise $+50\%$ increase of $\bar{\tau }_S(t)$, represented by red lines, and for a $-50\%$ decrease, represented by blue lines, relative to the basal state $\bar{\tau }_S=\bar{\tau }_S^b$. The dashed lines indicate the steady-state solutions of Eqs. (22). Top: Molecular concentration profiles across the normalized arterial thickness (along line AA’ in Fig. 2) for nitric oxide ($C_{\text {NO}}$, left) and reactive oxygen species ($C_{\text {ROS}}$, right). Middle: Average tissue nitric oxide concentration (left) and the corresponding variation in the radius–pressure relationship (right). Bottom: Distribution of smooth muscle cell (SMC) active stretches $\lambda _{\text {smc}}^a$ in the media layer of the arterial cross section resulting from the activation of NO-related chemical pathways (see Eq. (25)). Parameters’ values are listed in Table 2

### Local model: vascular tone regulation as a chemo-biological response

This section investigates the response of the chemo-mechano-biological local model alone. In particular, the effects of changes in the TAWSS by $\pm 50\%$ in relation to the baseline value are investigated, by varying $\bar{\tau }_S(t)$ linearly in time over a 2-minute interval along the slow time scale *t*. Referring to the flowchart depicted in Fig. 6, the simulations exclusively engage the local vessel model. There is no necessity for an internal loop on shear stresses, as these are *a priori* assigned and not obtained from the solution of the global cardiovascular model.

The obtained results are presented in Fig. 7. The dynamics of NO and ROS concentrations are prominently featured, revealing that even after a few minutes, the system has not yet reached a steady-state response. This observation is further supported by the geometrical dimensions and diffusion constants at hand, which provide a rough estimate of the transition time toward the steady-state values of approximately ${15\,\mathrm{\text {min}}}$, as given by $(\Delta _{S,0}^{\text {IM}}+\Delta _{S,0}^{\text {A}})^2/(2 D_{\text {NO}})$, (Carr, 2017).

Variations in the concentrations of NO within the arterial tissue give rise to changes in the contraction state of SMCs, resulting in substantial alterations in the unloaded configurations of arteries and in their pressure–radius relationship.

### Full model: system equilibrium and effect of a local alteration

The full framework, encompassing the global–local coupling, is addressed here. Constant global hemodynamic input conditions, corresponding to a basal heart frequency $f_h^b$, are considered. Since nothing changes with the slow time scale, the algorithmic procedure in Fig. 6 allows us to determine the equilibrium state associated with the balance between global flow conditions and local vessel mechanical properties.

Outcomes obtained from two local vessel models are investigated and compared. The first model replicates the original arterial cross section presented in Fig. 2a-c. The second one is assumed to host a calcified sub-region in the media layer as shown in Fig. 2f and discussed in Sect. 2.4.1.

#### Basal and adaptive homeostasis

The model featuring the original cross section without calcifications demonstrates an equilibrium state aligning with the reference conditions and representing a basal homeostatic state. Specifically, the smooth muscle cells (SMCs) exhibit a consistent and unchanging active stretch equal to the basal value, that is $\lambda _{\text {smc}}^a=\lambda _{\text {smc}}^{a,b}$. Blood flow conditions mirror those presented in Fig. 5, maintaining TAWSS at a constant and basal level, i.e., $\bar{\tau }_{S}=\bar{\tau }_{S}^b$, for each heartbeat. Consequently, NO and ROS are produced at basal levels within the intima, resulting in constant distributions $C_{\text {NO}}=C_{\text {NO}}^b$ and $C_{\text {ROS}}=C_{\text {ROS}}^b$ within the arterial wall.

**Fig. 8:**
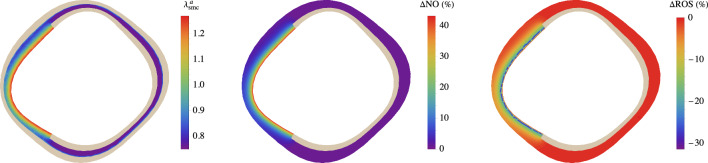
Outcomes from the full model showing the impact of calcification in the local vessel model (Sect. 3.2). Left: Distribution of the active stretch $\lambda _{\text {smc}}^a$ of smooth muscle cells (SMCs) within the (healthy) media. Center: normalized variation of NO concentration with respect to the basal value $\Delta \text {NO}=C_{\text {NO}}/C_{\text {NO}}^b-1$. Right: normalized variation of ROS concentration with respect to the basal value $\Delta \text {ROS}=C_{\text {ROS}}/C_{\text {ROS}}^b-1$. All plots are demonstrated in the zero-pressure configuration. The parameters’ values used in the analysis are listed in Tables 2 and 3

The scenario changes significantly when employing the local vessel model with calcifications. As depicted in Fig. 8, the active stretch of SMCs across the vessel wall shows significant non-uniformity in this new state. Certain regions exhibit considerably greater relaxation compared to the uniform basal contraction observed in the non-calcified scenario. Such variations arise from the altered diffusivity and production of NO and ROS within the calcifications that lead high inhomogeneities in the molecular distributions. Concentrations of molecular species vary by approximately $\pm 30\%$ compared to the non-calcified case.

**Fig. 9:**
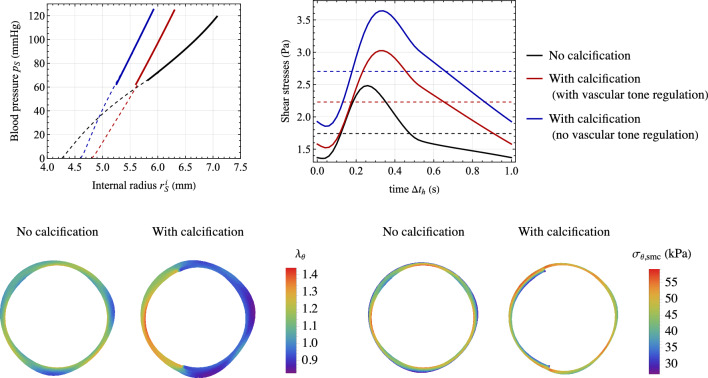
Outcomes from the full model showing the impact of calcification in the local vessel model (Sect. 3.2). Top: Pressure–radius relationships (left) and shear stresses over a heartbeat (right) for different scenarios: local vessel model without calcification, with calcification and vascular tone regulation, and with calcification without vascular tone regulation. Continuous lines in the pressure–radius plots depict pressure ranges experienced during a heartbeat, while the dashed sections represent relationships obtained unloading the vessel to zero pressure. Dashed lines in the shear stresses plot indicate Time-Averaged Wall Shear Stress (TAWSS). Bottom: Distribution of circumferential tissue stretch $\lambda _{\theta }=(\varvec{e}_{\theta } \cdot \textbf{C} \varvec{e}_{\theta })^{{1}/{2}}$ (left) and circumferential smooth muscle cells (SMCs) active stress $\varvec{\sigma }_{\theta ,\text {smc}}=(\varvec{e}'_{\theta } \cdot \varvec{\sigma }_{\text {smc}} \varvec{e}'_{\theta })^{{1}/{2}}$ where $\varvec{e}'_{\theta }$ is the unit vector derived from the push forward of $\varvec{e}_{\theta }$ (right). These plots illustrate the loaded configuration at Mean Arterial Pressure (MAP) and address the local model without calcification, as well as with calcification and vascular tone regulation. The parameters’ values used in the analysis are listed in Tables 2 and 3

As demonstrated in Fig. 9, alterations in the mechanical properties of the calcified arterial cross section significantly affect the pressure–radius relationship of the vessel. Notably, these changes stem from localized alterations in stress and strain distributions observed between the non-calcified and calcified scenarios.

Modifications in vessel pressure–radius mechanics induce changes in vessel compliance and resistance. A novel, non-basal, equilibrium state emerges from the global–local interplay. This demonstrates the capability of the computational framework in capturing adaptive homeostasis of the system. Remarkably, despite no alterations in the global hemodynamic input, the global–local coupling forecasts a $17\%$ increase in the systolic-diastolic peak compared to the non-calcified scenario (see Fig. 9), highlighting the relevance of the interplay between local alterations and global hemodynamic conditions.

#### Vascular tone regulation and shear stresses

Figure 9 also shows the pressure–radius relationship obtained without considering vascular tone regulation and hence maintaining a constant basal active stretch. The absence of vascular tone regulation leads to substantial variations in the vessel’s final mechanical response associated with calcifications, resulting in a notable increase in shear stresses. Specifically, the increase reaches $+55\%$ compared to $+28\%$ with vascular tone regulation, showcasing the significant impact of this regulatory mechanism.

### Full model: effect of global and local alterations

**Fig. 10:**
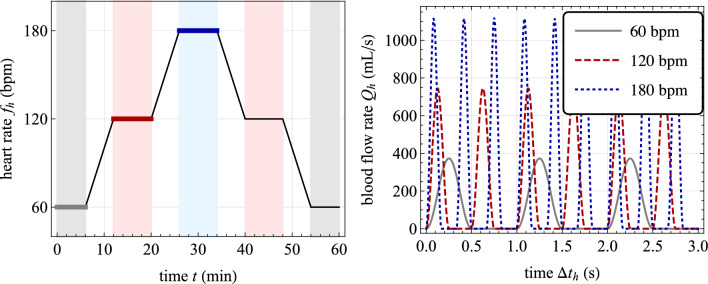
Outcomes from the full model showing the impact of increased cardiac frequency (Sect. 3.3). Left: Evolution of the cardiac frequency along the slow time scale *t*. Right: Blood flow profiles $Q_h(t_h)$ at cardiac frequencies $f_h^b$, $f_h^{a1}$ and $f_h^{a2}$ (as obtained from Eq. (31)) depicted relative to the fast time scale interval $\Delta t_h=t_h - \bar{t}$. Here, $\bar{t}$ represents time points of ${0\,\mathrm{\text {min}}}$, ${12\,\mathrm{\text {min}}}$ and ${26\,\mathrm{\text {min}}}$ for the basal heart rate (60 bpm), a mild increase (120 bpm), and a severe increase (180 pm) in cardiac frequency, respectively

In this section, the full model is used to simulate the impact of heightened blood flow on vascular tone regulation resulting from an alteration in heartbeats’ frequency. Three levels of cardiac frequencies are considered: the basal one $f_h^b$; a mild increase $f_h^{a1}=2 f_h^b$; and an intense one $f_h^{a2}=3 f_h^b$. The cardiac frequency varies along the slow time scale *t* within 60 min and follows the piecewise linear law shown in Fig. 10, where blood flow profiles respecting Eq. (31) are also shown for the three levels of cardiac frequencies.

**Fig. 11:**
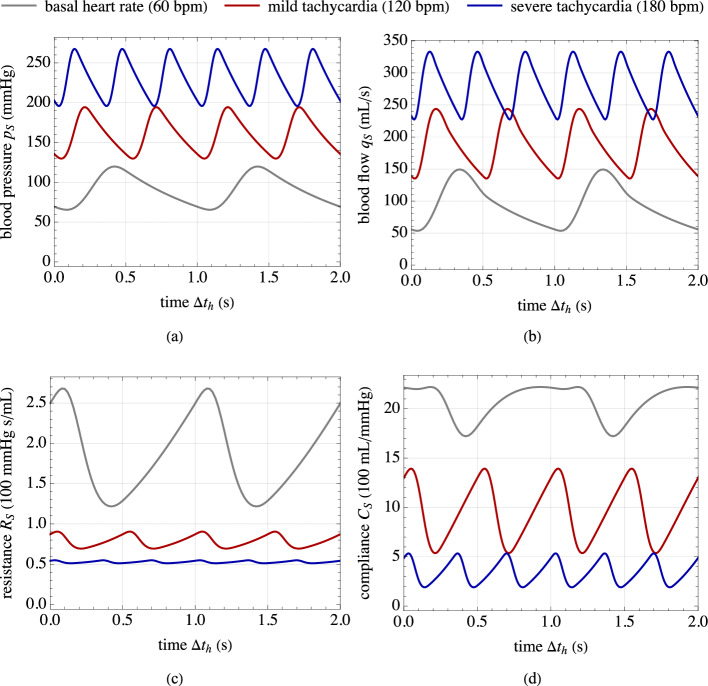
Outcomes from the full model showing the impact of increased cardiac frequency (Sect. 3.3). Temporal variations of hemodynamic outcomes within the segment of interest (see Fig. 10), depicted relative to the fast time scale interval $\Delta t_h=t_h - \bar{t}$. Here, $\bar{t}$ represents time points of ${0\,\mathrm{\text {min}}}$, ${12\,\mathrm{\text {min}}}$ and ${26\,\mathrm{\text {min}}}$ for the basal heart rate, a mild increase, and a severe increase in cardiac frequency, respectively. Results refer to: **a** blood pressure $p_S$; **b** blood flow $q_S$; **c** vessel resistance $R_S$; and **d** vessel compliance $C_S$. The parameters’ values used in the analysis are listed in Tables 2 and 3

**Fig. 12:**
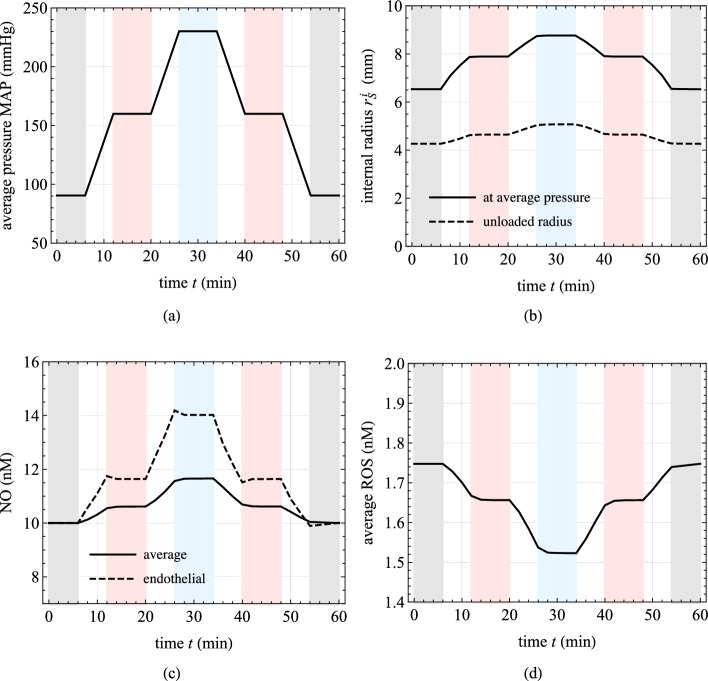
Outcomes from the full model showing the impact of increased cardiac frequency (Sect. 3.3). Temporal variations of chemo-mechano-biological outcomes within the segment of interest (see Fig. 10), depicted relative to the slow time scale *t*. Results refer to: **a** the average pressure during a cardiac cycle; **b** the internal radius $r_S^i$ in the loaded configuration at average pressure and in the unloaded configuration; **c** endothelial nitric oxide (NO) concentration $C_{\text {NO},e}$ and average concentration within tissue domain; **d** average concentration of reactive oxygen species (ROS) within tissue domain. The parameters’ values used in the analysis are listed in Tables 2 and 3

The plot in Fig. 11 illustrates the changes in hemodynamic conditions within the segment of interest across the three different frequencies in the fast time scale $t_h$. As depicted in Fig. 11a and b, the intensified heartbeat rate leads to a noteworthy surge in both blood pressure and blood flow. Notably, the proposed multiscale framework predicts distinct values of segment resistance and compliance for each frequency level (Figs. 11c,d), with high blood pressure triggering a substantial reduction in their values.

The reason behind this outcome is clear when examining Fig. 12, which demonstrates the system’s adaptation in the slow time scale *t*. As the heartbeat rate increases, it leads to a subsequent rise in mean blood pressure (Fig. 12a) and, consequently, an expansion of the vessel radius at the average pressure (Fig. 12b). The decrease in vessel resistance can be directly attributed to the obtained lumen area enlargement from Eq. (1), while the decrease in compliance arises from the nonlinear stiffening of the tissue, as evident from the slope of the pressure–radius curves at high pressures in Fig. 7.

The chemo-biological response of the local model is activated by the altered flow conditions, as emphasized in Figs. 12c and 12d. These figures illustrate the progression of endothelial production of NO, along with the subsequent variations in average NO and ROS concentrations. Consequently, the increased levels of NO lead to the active dilation of SMCs, as evidenced by the increase in the unloaded radius illustrated in Fig. 12b. The effect and underlying rationale of this mechanism are further explored in the next Sect. 3.3.1, where a comparative analysis is conducted.

#### Cardiovascular response and endothelial dysfunction

The predicted cardiovascular response of a vessel segment equipped with a responsive endothelium is contrasted with the response observed in the presence of mild and severe endothelial dysfunction, as introduced in Sect. 2.4.1.

**Fig. 13:**
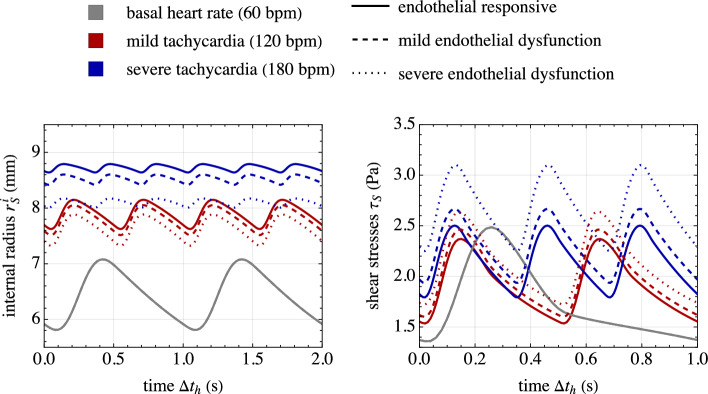
Outcomes from the full model showing the impact of increased cardiac frequency and endothelial dysfunction (Sect. 3.3.1). Temporal variations of internal radius $r_S^i$ (left) and shear stresses $\tau _S$ (right) within the segment of interest. The results are shown relative to the fast time scale interval $\Delta t_h=t_h - \bar{t}$, where $\bar{t}$ represents time points of ${0\,\mathrm{\text {min}}}$, ${12\,\mathrm{\text {min}}}$, and ${26\,\mathrm{\text {min}}}$ for the basal heart rate, a mild increase, and a severe increase in cardiac frequency, respectively. The parameters’ values used in the analysis are listed in Tables 2 and 3

Figure 13 shows the corresponding variations in the internal radius in the fast time scale $t_h$ across the three different cardiac frequencies. Notably, the impact of endothelial dysfunction on radius variations is evident. The most significant changes occur when transitioning from mild to severe disease levels and from mild to intense increase in cardiac frequency. These alterations in vessel radii have a direct effect on shear stresses, as also shown in Fig. 13. While shear stress values increase dramatically with cardiac frequency in the presence of severe endothelial dysfunction, the rise is only moderate or minor when endothelial functioning is better.

**Fig. 14:**
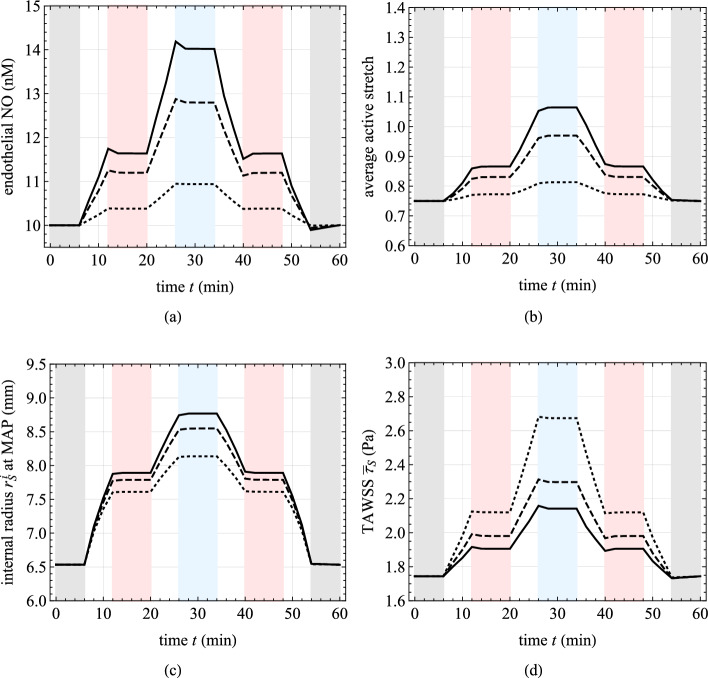
Outcomes from the full model showing the impact of increased cardiac frequency and endothelial dysfunction (Sect. 3.3.1). Temporal variations of chemo-mechano-biological outcomes within the segment of interest. Results, depicted relative to the slow time scale *t*, refer to: **a** endothelial nitric oxide (NO) concentration $C_{\text {NO},e}$; **b** average active stretch $\lambda _{\text {smc}}^a$ within the domain of the media layer in the tissue; **c** internal radius $r_S^i$ in the loaded configuration at the average pressure within the cardiac cycles; **d** time-averaged wall shear stresses TAWSS $\bar{\tau }_s$. The parameters’ values used in the analysis are listed in Tables 2 and 3

**Fig. 15:**
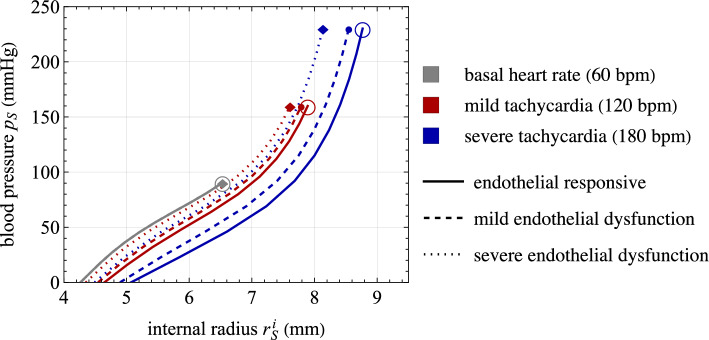
Outcomes from the full model showing the impact of increased cardiac frequency and endothelial dysfunction (Sect. 3.3.1). Relationship between blood pressure $p_S$ and internal radius $r_S^i$ from the unloaded configuration up to the average pressure within cardiac cycles. The parameters’ values used in the analysis are listed in Tables 2 and 3

These outcomes are further illustrated in Fig. 14, which highlights the local effect of endothelial dysfunction on the chemo-mechano-biological adaptation of the cardiovascular system related to an increase in cardiac frequency along with the slow time scale *t*. The increase in NO production with cardiac frequency is high for a responsive (intact) endothelium and decreases with the severity of endothelial dysfunction (Fig. 14a). Thus, the increase of the average active stretch in the tissue (Fig. 14b) and of the internal radius at mean pressure (Fig. 14c) associated with an increase of the cardiac frequency are highly affected by endothelial dysfunctions. Overall, severe levels of dysfunction lead to circa $50\%$ increase in TAWSS, while chemo-biological responsiveness reduces this increment to approximately $25\%$ (see Fig. 14d). The effects of the chemo-mechano-biological coupling are also depicted in Fig. 15, which illustrates the resulting radius–pressure relationship (up to the mean pressure obtained during the corresponding cardiac cycles) for the different investigated cases. Hence, this case study highlights the model capability in capturing the complex adaptive processes behind the regulation of the homeostatic equilibrium state in cardiovascular biomechanics, as affected by both global and local alterations.

## Conclusion

In this study, a computational model has been presented, which integrates chemo-mechano-biological pathways with cardiovascular biomechanics to investigate the regulation of vascular tone. By bridging the gap between global hemodynamics, local chemo-biological pathways, and arterial biomechanics, insights on the complex interplay between mechanical and biochemical factors in vascular tone regulation are achieved.

The findings highlight the critical role of vascular tone in maintaining cardiovascular homeostasis and its impact on overall circulatory function. Through the integration of detailed finite element analyses and reduced-order modeling of global hemodynamics, the ability to capture the intricate behaviors of arterial tissues in response to mechanical stimuli is demonstrated. By incorporating the diffusion–reaction mechanisms regulated by local hemodynamic conditions, the model offers insights into the molecular pathways involved in the regulation of smooth muscle cell contraction and relaxation.

The model’s response has demonstrated its capability to accurately represent the mechanical behavior of tissues and vascular segments and its variation with vascular tone (see Fig. 4). Additionally, the obtained values of NO concentration in the different case studies align well with the average values reported both in vivo (Sena et al., 2018) and *in vitro* (Hall and Garthwaite, 2009). As regards the global–local coupling, values of compliance and resistance of vascular segments obtained from finite element simulations at the local scale agree with the established literature and *in vivo* data (Korakianitis and Shi, 2006; Tossas-Betancourt et al., 2020; Kornet et al., 1998; Bank et al., 1995), demonstrating the effective transfer of information between global and local problems.

Simulation results obtained from the computational framework shed light on the effects of both local and global alterations on vascular tone adaptation. These alterations induce changes in local flow conditions and mechanical stimuli, thereby affecting chemo-biological pathways. Consequently, adjustments in vascular tone significantly impact wall shear stresses and intramural stresses and strains. These findings are crucial for comprehending cardiovascular responses to multifactorial stimuli and incorporating the role of adaptive homeostasis in *in silico* biomechanics frameworks (Davies, 2016). Prospectively, they hold promise for enhancing diagnostic and therapeutic strategies for cardiovascular diseases.

### Context of use and clinical significance

The developed simulation framework has highlighted the pivotal role of vascular tone regulation in the reliability of WSS predictions—a crucial aspect increasingly acknowledged as a reliable risk indicator in diverse cardiovascular pathologies (Gallo et al., 2016; Mazzi et al., 2022). Current computations of WSS-related indices typically assume vascular segments to be passive structures. However, integrating their active behavior, especially considering local vessel features like intramural calcifications, could significantly enhance their long-term predictive accuracy.

Endothelial dysfunction is closely associated with various cardiovascular pathologies such as atherosclerosis, hypertension, and diabetes (Marti et al., 2012). Yet, clarifying the mechanistic link between endothelial dysfunction and these pathologies warrants further investigation. In this regard, the proposed computational model serves as an effective tool, offering novel quantitative insights into the escalation of wall shear stress associated with endothelial dysfunction.

Furthermore, the model’s ability to characterize ROS concentrations within arterial tissues, dependent on NO production, presents a crucial avenue. Disrupted redox homeostasis can have detrimental effects on cells by disrupting signaling pathways or causing oxidative damage to essential biomolecules like proteins, lipids, and nucleic acids (Marti et al., 2012; Lacolley et al., 2017). Although numerous additional chemical pathways should be incorporated for a thorough characterization, future advancements hold the promise of a more comprehensive understanding of oxidative stress within biomechanical models of cardiovascular structures. This includes, for instance, understanding how oxidative stress influences cell–cell signaling pathways related to hypertension-induced vessel remodeling (Green et al., 2017; Wilstein et al., 2018).

Overall, this model could serve as a foundation to validate whether the incorporation of chemo-biological pathways in vascular tone regulation enhances the efficacy of *in silico* biomechanical models as predictive tools for assessing cardiovascular pathologies in clinical settings. However, several challenges and opportunities for future research remain.

### Limitations and future works

Enhancements in the description of molecular biology and biomechanics at various scales would significantly bolster the predictive capabilities of the model. Section 3.2 demonstrates the framework’s capacity to correlate vascular tone regulatory mechanisms with detailed local histological and geometrical properties, such as the distribution of calcifications. Integrating patient-specific data could yield various applications, assessing whether incorporating chemo-biological pathways in vascular tone regulation improves the predictive efficacy of *in silico* biomechanical models for evaluating cardiovascular pathologies in clinical settings. The model’s multiscale approach and use of the finite element method at the local level render it readily adaptable.

However, the cross-correlation between model parameters (and uncertainties) is instrumental to highlight possible nonlinearities and predict responses in a patient-specific setting. To this end, an extensive (global) sensitivity analysis would be necessary (Hamdia et al., 2019), possibly adopting techniques based on model order reduction and/or surrogate modeling (Urrea-Quintero et al., 2021).

Moreover, for more realistic applications, there is a need to expand the local model from 2D to 3D, as well as to encompass multiple active segments within the cardiovascular tree. This expansion may introduce additional computational costs, yet it is important to note that the procedure remains cost-effective within the current framework. The most resource-intensive simulation, associated with the case study involving varying cardiac frequencies, takes approximately 10 min on a standard PC laptop (Intel Core i7, 16 GB RAM). The remaining case studies require at most 1-2 min per simulation. Consequently, scalability remains achievable even when dealing with several segments of interest, especially considering that each segment can be run in parallel. Coupling between different segments occurs solely at the global cardiovascular level, which incurs minimal costs, even when managing numerous segments.

Further exploration into the roles of additional biochemical factors, signaling pathways, nervous system, and baroreceptors in vascular tone regulation is essential for a more comprehensive understanding of this intricate process. When incorporating mechanisms through which the cardiovascular system adapts to varying metabolic needs, the proposed approach might contribute to the study of pathologies related to inadequate blood supply to tissues during increased demand, such as during physical exercise. However,  although physical exercise leads to an increase in cardiac frequency, Sect. 3.3 provides only a partial representation of this phenomenon. A comprehensive understanding demands an investigation that integrates the autonomic nervous system, hormonal mechanisms, and variations in peripheral resistance (Green et al., 2017).

Additionally, Sect. 3.3.1 simulates endothelial dysfunction by reducing the sensitivity of endothelial cells to shear stress. However, this represents just one aspect of the disorder, which can stem from various sources leading to reduced bioavailability of endothelium-derived relaxing factors—be it impaired production capabilities or increased production of contracting factors (Marti et al., 2012), as well as altered transport of macromolecules to and from the tissues and blood (Ray et al., 2023).

Moreover, the present paper neglects contraction dynamics of actin-myosin bridges. In fact, active contraction of SMCs follows an increased cytosolic concentration of $Ca^{2+}$ that initiates a change in the chemical state of myosin and leads to phosphorylation of myosin heads and attachment to actin. The major effect of this chain of events can be modeled by introducing a functional dependency on the actin-myosin state of SMCs stiffness and maximum stress [parameters $C_{\text {smc}}$ and $P_{\text {smc}}^{max}$ in Eq. (16)]. Such an effect would not change the main outcomes analyzed in this work and is therefore not included for the sake of the model’s parsimony. However, the developed framework is readily generalizable to account for more refined descriptions, see e.g., Zulliger et al. (2004); Stålhand et al. (2011); Murtada et al. (2017). In fact, while calcium dynamics often interact with NO pathways and other signaling molecules in various diseases, in some specific pathological conditions, alterations in calcium signaling within smooth muscle cells might be more prominent than direct involvement of NO pathways in disease manifestation (such as Calcium Channelopathies).

Furthermore, the current model would benefit from more refined fluid–structure interaction (FSI) approaches to better capture the dynamic interactions between blood flow and arterial wall mechanics. The inclusion of more sophisticated FSI techniques would provide a more accurate representation of the complex fluid–structure interactions occurring within the arterial system. In addition, the presented computational model focuses on specific arterial segments and does not account for the complexity of arterial tree branches. The extension of the model to include a more comprehensive representation of the arterial network, including the branching geometry and heterogeneity of different arterial segments, would enhance its realism and applicability to a wider range of physiological scenarios.

Finally, the limited number of arterial segments considered in the global cardiovascular model hinders depicting complex wave reflections and diverse blood flow distribution between organs. A more comprehensive model with additional segments and diverse branches could enhance the range of applications of present framework (Quarteroni et al., 2016). Moreover, the scope of the model could be expanded to include distributed 1D network models for cardiovascular biomechanics. Incorporating such network models would enable the study of hemodynamic effects and chemo-biological interactions in a broader context, considering the interplay between different arterial segments and their collective impact on global cardiovascular function. In fact, 0D cardiovascular models offer insights into overall hemodynamics but struggle to capture detailed phenomena like spatial variations in parameters such as pulse wave velocity (PWV) along the arterial tree.

Despite these limitations, the presented computational model serves as a valuable tool for advancing our understanding of vascular tone regulation and its implications for cardiovascular health since coupling, for the first time, a plethora of multi-factorial mechanisms at different time and length scales. Continued research in this field will deepen our knowledge and open new avenues for exploring the interplay between mechanical and biochemical factors in vascular physiology.

## Figures and Tables

**Fig. 16:**
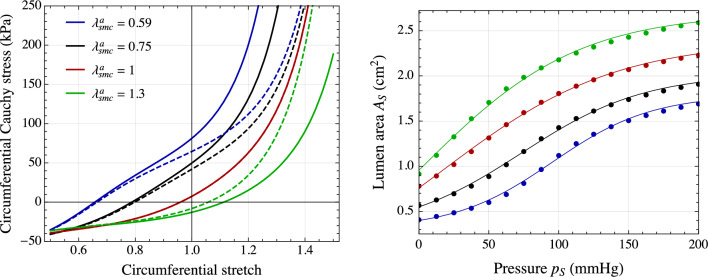
Left: circumferential stress–strain relationship in the media layer for a displacement-driven uniaxial test in the circumferential direction (with fixed axial stretch and radial stretch from the incompressibility condition). Results are obtained with different values of active stretches $\lambda _{\text {smc}}^a$, and considering the mechanism of inelastic collagen straightening (with $\xi _{\text {c}}=0.15$, continuous lines) or not (with $\xi _{\text {c}}=0$, dashed lines). Right: relationship between pressure $p_S$ and lumen area $A_S$ at different levels of SMC active stretch $\lambda _{\text {smc}}^a$ (imposed uniformly within the media layer). Numerical outcomes from finite-element simulations of the local model mechanics (symbols) are compared with the interpolated responses fitted via Eq. (27) (continuous lines, best-fit values of parameters in Table 4). Value of parameters, if not differently specified, are given in Table 2

## Data Availability

Data sets generated during the current study are available from the corresponding author on reasonable request. During the preparation of this work, the authors utilized ChatGPT to enhance the spelling, grammar, and clarity of the abstract and contribution sections. Following the use of this tool, the authors thoroughly reviewed and edited the content as necessary and assume full responsibility for the publication’s content. The authors recognize the potential benefits and limitations of using generative AI and AI-assisted technologies in research and have taken measures to ensure that the use of these technologies is transparent and ethical.

## Appendix A: Generation of arterial cross-section geometry

In a Cartesian system parametrized in coordinates (*x*, *y*) and denoting by $\theta (x,y)=\arctan (y/x)$, domain $\Omega _{\text {M},0}$ of the media layer is built as the region comprised between the intima layer $\Sigma _{\text {I},0}$, described as:

A.1 $$\begin{aligned} \Sigma _{\text {I},0} = \Big \{(x,y) \;\; \text {s.t.}\; \; x^2+y^2= \big (R_{\text {I}}(x,y)\big )^2 \; \; \text {with} \;\; R_{\text {I}}(x,y) = R_{s,0}^i + \Delta _{s,0}^{\text {IM}} \eta _{\text {I}} \cos \left( \gamma _I \theta (x,y)- \theta _I\right) =0\Big \}\,, \end{aligned}$$

and the media-adventitia boundary, reading:

A.2 $$\begin{aligned} \Sigma _{\text {M},0} = \Big \{(x,y) \;\; \text {s.t.} \; \; x^2+y^2= \big (R_{\text {M}}(x,y)\big )^2 \; \; \text {with} \;\; R_{\text {M}}(x,y)=R_{\text {I}}(x,y) + \Delta _{s,0}^{\text {IM}} \left[ 1 + \eta _\text {M}\cos \left( \gamma _\text {M}\theta (x,y) - \theta _\text {M}\right) \right] =0\Big \}\,. \end{aligned}$$

Analogously, domain $\Omega _{\text {A},0}$ for the adventitia layer is built as the region comprised between the media-adventitia boundary $\Sigma _{\text {M},0}$ and the outer adventitia layer:

A.3 $$\begin{aligned} \Sigma _{\text {A},0} = \Big \{(x,y) \; \; \text {s.t.} \; \; x^2+y^2= \big (R_{\text {A}}(x,y)\big )^2 \; \; \text {with} \;\; R_{\text {A}}(x,y)=R_{\text {M}}(x,y) + \Delta _{s,0}^{\text {A}} \left[ 1+ \eta _\text {A}\cos \left( \gamma _\text {A}\theta (x,y) - \theta _\text {A}\right) \right] =0\Big \}\,. \end{aligned}$$

Here $R_{s,0}^i$ is the mean reference internal radius, $\Delta _{s,0}^{\text {IM}}$ the mean intima-media thickness, and $\Delta _{s,0}^{\text {A}}$ the mean adventitia thickness. Moreover, $\eta _{*}$, $\gamma _{*}$ and $\theta _{*}$ are shape-governing parameters introduced to deviate from a perfectly cylindrical geometry (with $*=\text {I},\text {M},\text {A}$). For obtaining the cross-section geometry employed in numerical applications, the following values are adopted: $\eta _{\text {I}}=\eta _\text {M}=\eta _\text {A}=1/4$ $\gamma _{\text {I}}=4$, $\gamma _\text {M}=\gamma _\text {A}=3$, $\theta _{\text {I}}=0$ $\theta _\text {M}=\pi /3$ and $\theta _\text {A}=\pi /6$.

## Appendix B: Additional results on arterial local model

The obtained stress–strain relationship obtained from Eq. (17) in the media layer is reported in Fig. 16. Results refer to a displacement-driven uniaxial test in the circumferential direction, with fixed axial stretch and radial stretch from the incompressibility condition. Different values of active stretches $\lambda _{\text {smc}}^a$ are considered, and the effect of inelastic collagen straightening is also investigated.

The effectiveness of the surrogate modeling approach for the description of the relationship between pressure $p_S$ and lumen area $A_S$ from finite-element simulations of the local model mechanics is investigated in Fig. 16. The response is obtained for different levels of SMC active stretch $\lambda _{\text {smc}}^a$ (imposed uniformly within the media layer), and the corresponding best-fit values of parameters are reported in Table 4.

**Table 4:** Best-fit values of parameters for the surrogate model $A_S=A_S(p_S)$ in Eq. (27) for different values of SMC active stretch $\lambda _{\text {smc}}^a$

	a ($\textrm{cm}^{2}$)	b (−)	c (mmHg)	d $(\textrm{cm}^2)$
$\lambda _{\text {smc}}^a= 0.59$	1.483	2.696	95.625	0.306
$\lambda _{\text {smc}}^a= 0.75$	1.749	1.669	73.509	0.273
$\lambda _{\text {smc}}^a= 1$	2.864	0.274	17.008	$-0.476$
$\lambda _{\text {smc}}^a= 1.3$	3.463	0.013	0.748	$-0.765$
